# Biomimetic bone calcium phosphate-based scaffolds fabricated via ceramic vat photopolymerization: Effect of porosity, sintering temperature, mineralogical phases and trace elements on the osteogenic potential

**DOI:** 10.1016/j.mtbio.2026.103074

**Published:** 2026-03-26

**Authors:** Antonia Ressler, Roope Ohlsbom, Virginia Alessandra Gobbo, Markus Hannula, Katharina Keck, Harish Swaminathan, Toni-Karri Pakarinen, Mehdi Mohammadi, Jari Hyttinen, Jonathan Massera, Martin Schwentenwein, Erkka J. Frankberg, Erkki Levänen, Arjen Gebraad, Susanna Miettinen

**Affiliations:** a Faculty of Engineering and Natural Sciences, Tampere University, Korkeakoulunkatu 6, P. O. Box 589, Tampere, 33014, Finland; b Faculty of Medicine and Health Technology, Tampere University, Arvo Ylpön katu 34, Tampere, 33520, Finland; c Tays Research Services, Wellbeing Services County of Pirkanmaa, Tampere University Hospital, Elämänaukio, Kuntokatu 2, Tampere, 33520, Finland; d Tampere University Hospital, Elämänaukio, Kuntokatu 2, Tampere, 33520, Finland; e Lithoz GmbH, Mollardgasse 85a/2/64-69, Vienna, 1060, Austria

## Abstract

In response to the growing demand for novel approaches in bone repair, scaffolds that mimic natural bone microstructure and mineralogical composition were developed using a ceramic vat photopolymerization (VPP) method. Due to varying reported results regarding appropriate microstructural characteristics, this study aimed to clarify the best pore size distribution and porosity among the tested scaffolds for an efficient osteogenic response. Scaffolds based on hydroxyapatite both support new bone formation by osteoblasts and can be resorbed by osteoclasts. An average pore size of ∼400 μm and porosity of 45.61% showed the best mechanical properties and osteogenic response, allowing cell penetration, and supporting cell-cell interactions and the differentiation process. When Sr,Mg,Zn-substituted hydroxyapatite is used for scaffold fabrication, the required high sintering temperatures lead to the transformation of hydroxyapatite into *β*-tricalcium phosphate, a common calcium phosphate used in bone tissue engineering. However, the new mineralogical phase results in different surface properties that do not support appropriate cell attachment on scaffolds with higher negative surface charge and lower wettability. This work emphasizes the potential of ceramic VPP in the development of biomimetic scaffolds that mimic natural bone tissue and provides guidelines on which microstructural characteristics are appropriate for efficient bone regeneration. This study also raises new questions regarding cell attachment on *β*-tricalcium phosphate-based scaffolds, which are currently under exploration.

## Graphical abstract

**Figure:**
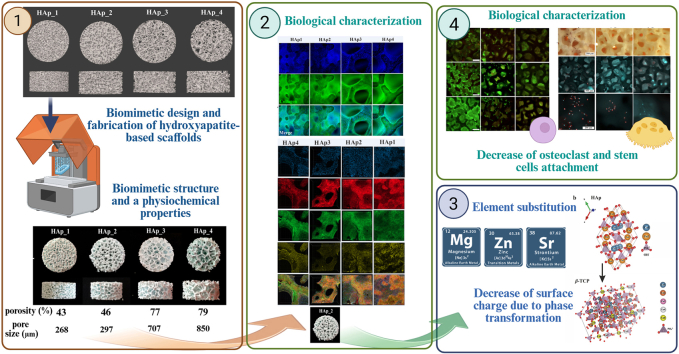

## 1 Introduction

Bone grafting using autografts, allografts, and bone graft substitutes (scaffolds) is a surgical procedure widely used for regeneration of bone fractures and bone defects caused by trauma and bone-related diseases. It is the second most common tissue transplantation procedure, after blood transfusions, with more than 2 million bone grafts performed annually worldwide [1]. Bone grafting involves placing bone or artificial biomaterial into the bone defect to allow and enhance the regenerative process when bone defects exceed a critical sized defect of 2 cm [[2], [3], [4]]. Autologous and allograft grafts are considered a gold standard in surgical procedures for bone regeneration. However, they are associated with several limitations such as a long bone regeneration process, the constrained amount of tissue available from donor areas, donor area bleeding, nerve damage, surgical complications, and high costs, urging the development of alternative synthetic bone grafts [5,6]. Furthermore, with an aging global population, the incidence of bone-related morbidities is increasing, resulting in even greater demand for development of novel bone grafts. According to the World Health Organization, the proportion of the world's population over 60 years will nearly double from 12% to 22% between 2015 and 2050 [7]. As result, global bone graft market is expanding with annual growth rate of ∼7% for the period of 2021-2030, projected to reach a market value of $4.60 billion by 2030 [[8], [9], [10], [11]]. The growth is not only driven by aging population, but also by technological advancements in bone tissue engineering that have enabled the treatment of wider range of bone defect and diseases [12].

The development of advanced scaffolds remains a significant challenge due to complex biological and mechanical requirements that need to be addressed for adequate bone regeneration [13]. An optimal scaffold for bone regeneration should provide temporary mechanical stability to preserve the structural integrity of the defect site and surrounding tissues during the healing process. As a bioactive and regenerative-compatible material, it should actively promote osteogenesis, while, at the same time, allow resorption by osteoclasts to ensure that the graft is gradually replaced by newly formed bone tissue. Obtaining an optimal scaffold by mimicking the delicate mechanical characteristics, structural complexities, and biological functions of bone tissue remains a significant challenge to this date [14,15]. New cutting-edge additive manufacturing technologies are allowing researchers to get closer to the optimal structural design. Additive manufacturing can be classified into several categories: powder bed fusion, direct energy deposition, binder jetting, sheet lamination, material extrusion, material printing, and vat photopolymerization (VPP). Compared with other fabrication methods, VPP offers several key advantages, including high dimensional accuracy, smooth surface finish of the printed parts, and minimal need for post-processing. In addition, it enables the production of dense ceramic components and provides clear benefits in manufacturing intricate, micro-scale geometries. These capabilities make VPP particularly well suited for processing high-performance structural ceramic components with complex architectures [16]. Ceramic VPP has shown significant promise in the fabrication of bone scaffolds with intricate micro-architectures and complex shapes, closely mimicking the natural bone structure [[17], [18], [19], [20], [21]]. The VPP technique relies on a computer-aided design (CAD) file of the desired structure, which is digitally sliced into a series of cross-sectional layers. A three-dimensional (3D) object is constructed by sequentially fabricating these layers. Each thin layer of a photocurable resin, consisting of photopolymerizable monomers, is continuously exposed to laser irradiation to induce photopolymerization. As a result, the liquid resin solidifies layer by layer, ultimately forming the complete 3D structure [22]. Due to its layer-by-layer fabrication principle, VPP allows precise fabrication of patient-specific scaffolds that match the patient's unique defect structure opening the potential for personalized scaffolds' design. However, the ideal scaffold microstructure for promoting functional bone regeneration in terms of pore size distribution, pore shape and porosity, remains an open question under active investigation [20,21,23].

Hydroxyapatite (HAp, Ca_10_(PO_4_)_6_(OH)_2_) is the main inorganic component of natural bone tissue and is commonly used as a biomaterial for bone tissue engineering applications. It promotes osteogenic activity and osteointegration properties, along with biocompatibility and gradual biodegradability through osteoclastic resorption [24]. In our previous study, ceramic VPP was used to fabricate a biomimetic bone scaffold with a porosity and pore size distribution that closely resemble the morphology of cancellous bone tissue [20,21]. HAp substituted with strontium (Sr^2+^), magnesium (Mg^2+^) and zinc (Zn^2+^) ions was used as a starting powder. Trace elements were introduced in the HAp lattice to mimic chemical composition of natural bone tissue and to enhance osteoinductive properties [20,25]. Sr^2+^, Mg^2+^ and Zn^2+^ ions were selected due to their unique effect in bone regeneration processes where (i) Sr^2+^ enhances the expression of bone related markers, (ii) Mg^2+^ ions function as growth factors during the initial stages of osteogenesis and bone growth, while (iii) Zn^2+^ ions play a vital role in alkaline phosphatase (ALP) expression, creating an alkaline environment conducive to the precipitation of calcium phosphates (CaP) and the mineralization of the extracellular matrix (ECM) [[26], [27], [28], [29], [30]]. After sintering, *β*-tricalcium phosphate (*β*-TCP) was detected in addition to HAp. This phase transformation is attributed to the presence of trace elements, which can destabilize the HAp structure and promote the formation of *β*-TCP [20].

The aim of the present study was to (i) investigate the potential of ceramic VPP for scaffold fabrication, (ii) assess how surface properties influence cell behaviour, (iii) identify the most suitable scaffold microstructure among the tested variations, and (iv) explore the effect of ion incorporation on cellular responses. The main research question that the present study aims to answer is which combination of sintering temperature, scaffold microstructural characteristics (porosity, pore size, and interconnectivity), and ion incorporation yields the most favourable biological performance: optimal cell attachment, cell-cell interactions, and osteogenic response in the fabricated scaffolds. First, the optimal sintering temperature of the HAp biomaterial was evaluated by examining its effects on osteoclast attachment and ion exchange. Furthermore, recognizing the critical role of scaffold microstructure in the bone regeneration process, four distinct CAD models differing in porosity and pore size distribution were designed and fabricated using ceramic VPP to produce biomimetic scaffolds based on non-substituted HAp. The effect of different scaffold microstructures on mechanical properties and osteogenic response was investigated. After an appropriate scaffold design was determined, the impact of the Sr,Mg,Zn-substitution ions (1 and 5 mol%) on osteogenesis, osteoclast formation, and osteoclastic resorption of the material was investigated, as well as changes in material characteristics determining the biological response. This study provides valuable insights into the best scaffold microstructure among the tested variations required for effective bone regeneration. The findings not only expand the current capabilities of ceramic VPP in bone tissue engineering but also highlight key mechanistic questions that must be addressed to fully harness the potential of biomimetic engineering obtained by ceramic VPP.

## 2 Materials and methods

### 2.1 Substituted HAp powder and ceramic slurry preparation

Multi-substituted (Sr^2+^, Mg^2+^ and Zn^2+^) and non-substituted HAp powders were synthesized by the wet precipitation method as previously reported [20]. In brief, HAp powders were prepared by dissolving strontium nitrate (Sr(NO_3_)_2_), zinc nitrate hexahydrate (Zn(NO_3_)_2_ · 6H_2_O) and magnesium chloride hexahydrate (MgCl_2_ · 6H_2_O) in demineralized water at 40 °C, followed by addition of calcium oxide (CaO) at the same temperature. Subsequently, ammonium dihydrogen phosphate (NH_4_H_2_PO_4_) was added to solution to achieve a (Ca + Sr + Zn + Mg)/P molar ratio of 1.67, required for stoichiometric HAp. Stirring was continued for 10 h at 90 °C, followed by an overnight aging step at room temperature (RT). The selected (Sr + Zn + Mg)/(Ca + Sr + Zn + Mg) ratios were 0 (HAp), 1 (HAp_1MIX) and 5 (HAp_5MIX) mol% with equal proportions of substituted ions. After synthesis, the powders were filtered and dried at 90 °C. Powders were milled in ethanol using a planetary ball milling machine (Fritsch, Germany) for 2 h at a rotating speed of 200 rpm with zirconia milling balls of 0.5 cm in diameter, followed by drying for 2 h at 90 °C and sintering at 800 °C with a heating rate of 10 °C/min with dwell time of 2 h at the sintering temperature. Obtained powders were mixed with a commercial photocurable resin premix supplied by Lithoz GmbH in a volume % ratio of 38/62 as powder/resin.

### 2.2 CAD model, printing, debinding and sintering of HAp bioceramics

**Fig. 1:**
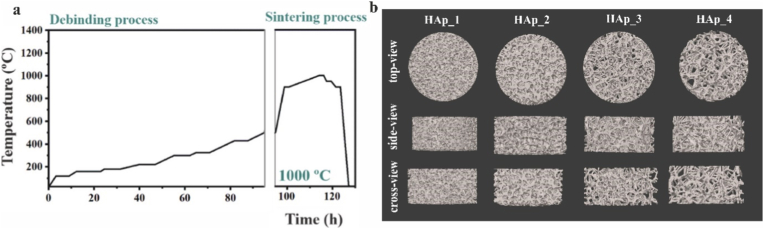
(a) Debinding and sintering schedule of the heat treatment process employed to obtain the final sintered porous structures according to our previous study [20]. (b) Top, side and cross-section views of the CAD model of scaffold with different porosities (HAp1, HAp2, HAp3 and HAp4) obtained by *nTopology* software. The stl. files of porous scaffolds are available at: https://doi.org/10.5281/zenodo.17120203.

To determine the best sintering temperature in the range of 900 – 1300 °C for effective cell attachment, a CAD model of a solid disc with dimensions of 3 mm (height) × 7 mm (diameter) was prepared using nTopology 3.35.2 software and exported as an .stl file to the slicer software of the 3D printer (Lithoz GmbH). Discs were fabricated using the CeraFab 7500 (Lithoz GmbH) DLP VPP printer with a layer thickness of 25 μm, and ceramic slurry based on non-substituted HAp at 45 °C. After printing, discs were cleaned by air brush and the commercial cleaning agent LithaSol 80 (Lithoz GmbH) was used to remove any uncured polymer resin. To eliminate the cured polymer, the as-fabricated structure underwent controlled thermal treatment (debinding), followed by sintering to achieve the desired mechanical properties in the final structure. The same debinding process was used for all samples, while sintering was performed at different temperatures (900, 1000, 1100, 1200, and 1300 °C) in a laboratory-scale muffle furnace (RHF1500, Carbolite Ovens ltd., UK) in air atmosphere (Fig. 1a). The debinding and sintering process used in our previous study was applied to the printed structures [20]. Samples were labelled as HAp_900, HAp_1000, HAp_1100, HAp_1200 and HAp_1300.

CAD models (Fig. 1b) of the desired structures that mimic highly porous natural bone tissue with different porosities were designed using nTopology 3.35.2 software and exported as an .stl file. The porous cylinder shape CAD models with outer dimensions of 3.5 mm (height) × 7 mm (diameter) were fabricated as previously described for discs materials. After initial cleaning with pressurized air, the scaffolds were immersed in LithaSol 80 and subjected to a low-power ultrasonic bath (5 min) at RT to ensure removal of any remaining uncured resin from the complex porous structures [21]. The cleaning process was repeated three times to ensure that the inner part of the structures was properly cleaned of the uncured resin. A slurry composed of non-substituted HAp was used for scaffold fabrication. The resulting scaffolds, exhibiting varying porosities, were labelled HAp1, HAp2, HAp3 and HAp4. Once the best microstructure among the tested specimens was established, further scaffold characterization was conducted using scaffolds fabricated from slurries prepared with HAp_1MIX (scaffold: HAp2_1MIX) and HAp_5MIX (scaffold: HAp2_5MIX) powders. Scaffolds made from non-substituted HAp were used as control. Debinding process for discs samples was also used for porous scaffolds, while sintering of the porous scaffolds was conducted only at 1000 °C under the same conditions as described for the discs.

### 2.3 Characterizations of fabricated scaffolds

#### 2.3.1 Microstructure analysis

The microstructure properties of the HAp1, HAp2, HAp3 and HAp4 scaffolds were analyzed using 3D *X*-ray microtomography (micro-CT) imaging, as described in our previous study [31]. In brief, the samples were imaged using the Xradia MicroXCT-400 device (Zeiss, Pleasanton, USA). A pixel size of 5.64 μm was achieved with a 4× objective. The *X*-ray tube was operated at 60 kV with a current of 166 μA, and a total of 1601 projections were captured, each with an exposure time of 3 s. The reconstructions were carried out using the XMReconstructor software, which employs the filtered back projection algorithm. A box measuring 3700 × 3700 × 400 μm was selected from each sample for analysis. Porosities, open porosity, wall thickness and pore size distributions were calculated using ImageJ with the BoneJ plugin [32]. Interconnectivity was assessed using a custom MATLAB algorithm described in Ref. [32]. The scaffolds were imaged using a digital camera in micro imaging mode to examine top, side, and cross-sectional views.

#### 2.3.2 Compression test

The obtained porous scaffolds (7 × 3.5 mm, at least n = 7) were subject to compression testing using a non-hydraulic universal material testing machine (Instron 5960, Instron Ltd.), as previously described in our study [20]. In brief, a constant applied displacement rate of 0.5 mm/min at RT (T = 23.0 °C ± 0.5) was used. The compressive load and displacement were recorded at 0.1 s intervals during testing with a cell load of 2 kN. Maximum compressive engineering stress in respect to the whole sample diameter was determined using the software associated with the testing machine (Bluehill 3).

#### 2.3.3 Zeta potential test

Zeta potential (ζ-potential) measurements were conducted using an electrokinetic analyzer (SurPASS3, Anton Paar), following the procedure described in our previous study [33]. Briefly, a 1 mM KCl solution (VWR International, Radnor, PA, USA) was prepared in ultrapure water (pH ∼ 5.5) to serve as the electrolyte. Samples (HAp2, HAp2_1MIX and HAp2_5MIX) were crushed using ceramic mortar and used in powder form to fill the powder holder in the Cylindrical Cell. The measurements were performed at physiological pH (7.40 ± 0.05), with the pH being adjusted by buffering the electrolyte with 0.05 M NaOH and eventually 0.05 M HCl solutions. The permeability index was set at 100 ± 10. Three measurement cycles were performed, each of them lasting approximately 90 s. A *β*-TCP scaffold was used as control sample. This scaffold was prepared using the CAD design HAp2 (Fig. 1b) and fabricated with the commercial slurry LithaBone TCP 300 (Lithoz GmbH) as described in the section 2.2.

#### 2.3.4 Contact angle measurements

Wettability of the fabricated discs HAp, HAp_1MIX and HAp_5 MIX sintered at 1000 °C was analyzed using contact angle analysis. Disc samples were prepared as explained in section 2.2 *CAD model, printing, debinding and sintering of HAp bioceramics*. The measurement was conducted on disc samples of 5 mm in diameter in static conditions using the sessile drop method (Attension Theta Lite tensiometer, Biolin Scientific, Stockholm, Sweden). A 3 μL drop of distilled water was placed on the surface of the experimental samples using a 1000 μL Hamilton syringe (Reno, NV, USA). The contact angle was analyzed from a recorded 10-s video using the in-built sessile drop program in OneAttension software (Biolin Scientific, Stockholm, Sweden). Due to time-dependent water droplet absorption, the contact angle was determined from the frame exported immediately after the droplet was positioned on the sample surface. The measurements were performed in triplicate, and the contact angle was determined from both the left and right sides of each droplet.

### 2.4 Biological characterization

#### 2.4.1 Human bone marrow-derived mesenchymal stem/stromal cells

##### 2.4.1.1 Isolation and characterization of human bone marrow-derived mesenchymal stem/stromal cells

Human bone marrow-derived mesenchymal stem/stromal cells (hBMSCs) were isolated from bone marrow aspirate samples obtained from a 70-year-old man (donor 1) and 67-year-old woman (donor 2), during a surgical procedure at the Coxa Hospital for Joint Replacement. The samples were collected with the patients’ written, informed consent and under the approval of the Ethics Committee of the Pirkanmaa Hospital District, Tampere, Finland (R15174). Isolation of hBMSCs was done as previously described [34]. Isolated hBMSCs were preserved in gas-phase nitrogen and thawed for characterization and experiments. The mesenchymal origin of the hBMSCs was confirmed through flow cytometric characterization of cell surface markers and assessment of their capacity for osteogenic and adipogenic differentiation. The cell characterization results are provided in the supplementary materials.

Cell surface marker expression of undifferentiated human bone marrow-derived mesenchymal stem/stromal cells (hBMSCs) was measured at passage 3 (donor 1) and passage 2 (donor 2) by flow cytometry (FACSCanto II; BD Biosciences, Franklin Lakes, NJ, USA), as previously described [35]. The results are presented in Supplementary Tables 1 and 2. For donor 1, the hBMSC population showed an expression profile typical for human mesenchymal stem cells (MSCs) cultured *in vitro*. For donor 2, the hBMSC population showed an expression profile typical for MSCs cultured *in vitro* for markers CD34, CD73, CD90, CD105, and HLA-DR [[35], [36], [37]]. CD14 and CD19, however, showed higher values than MSCs usually do. This could indicate that at least with passage 2 there were still some immune cells present as these markers are usually associated with immune cell functions [38,39].

Osteogenic (OD) and adipogenic differentiation (AD) of hBMSCs was performed as previously described [40]. For OD, hBMSCs were cultured for 21 days in osteogenic medium (OM), consisting of αMEM (Gibco, Thermo Fisher Scientific, Waltham, Massachusetts, USA), 5% human serum (Serana, Brandenburg, Germany), and 100 U/mL penicillin and 100 μg/mL streptomycin (Lonza, Basel, Switzerland) with osteogenic supplements 250 μM ascorbic acid 2-phosphate (Sigma-Aldrich, St. Louis, Missouri, USA), 10 mM-glycerophosphate (Sigma-Aldrich) and 5 nM dexamethasone (Sigma-Aldrich). For AD, hBMSCs were cultured for 21 days (donor 1) or 14 days (donor 2) in adipogenic medium, consisting of αMEM, 3 % human serum, and 100 U/mL penicillin and 100 μg/mL streptomycin with adipogenic supplements 1 μM dexamethasone, 100 nM insulin (Gibco, Thermo Fisher Scientific), 17 μM D-pantothenate (Sigma-Aldrich) and 33 μM biotin (Sigma-Aldrich). Controls were established by culturing hBMSCs in basic medium (BM) lacking osteogenic and adipogenic supplements. The culture medium was changed twice a week. Osteogenic potency of hBMSCs was assessed by deposition of CaP mineral, using Alizarin Red S staining as previously described [41]. Adipogenic potency of hBMSCs was assessed by detecting intracellular lipid accumulation with a Nikon Eclipse TE2000 S inverted phase contrast microscope equipped with a DS-5M camera (Nikon Corporation, Tokyo, Japan) using a 10 × objective. Supplementary Figs. 1 and 2 show that both donor-derived hBMSC cell lines can undergo OD and AD. Alizarin Red S staining showed that the hBMSCs produced CaP mineral deposits in osteogenic culture conditions, whereas the phase contrast images proved that adipogenic culture conditions led to accumulation of intracellular lipid droplets.

##### 2.4.1.2 3D culture of human bone marrow-derived mesenchymal stem/stromal cells

The scaffolds were sterilized using dry heat sterilization. Before cell seeding, the scaffolds were moved to hydrophobic 24-well plates (Nunc, Thermo Fisher Scientific), and incubated for 24 h in BM. The scaffolds were seeded with hBMSCs at passage 4. Cells were added on each scaffold as a single 10 μl droplet of cell suspension containing 4 × 10^4^ hBMSCs in BM or OM and incubated for 90 min in an incubator (37 °C, 5% CO_2_) to allow cell attachment and migration inside the scaffold. After the incubation, BM or OM was added to a final volume of 1 ml per well. Cell-seeded scaffolds were cultured up to 21 days in BM or OM in standard cell culture conditions (37 °C, 5% CO_2_). Medium was changed every 3 or 4 days.

##### 2.4.1.3 Qualitative analysis of cell viability

Qualitative analysis of cell viability (n = 2) was performed after 1 and 7 days of culture using Live/dead cell imaging kit (Invitrogen, Thermo Fisher Scientific, Waltham, Massachusetts, USA). After removing medium from the wells, the scaffolds were washed once with phosphate buffered saline (PBS) and incubated for 45 min at RT in PBS containing 0.5 μM Calcein-AM (CaAM) and 0.25 μM ethidium homodimer-1 (EthD-1). CaAM stains live cells green and EtHD stains dead cells red. After the incubation, the scaffolds were imaged with Leica DMi8 Inverted Microscope equipped with a K5 sCmOS microscope camera (Leica Microsystem GmbH, Wetzlar, Germany), using 5 × objective. Image processing was performed using Fiji ImageJ software (version 2.3.0).

##### 2.4.1.4 Proliferation assay

Quantitative analysis of cell number (n = 3) was performed on days 1 and 7 by analyzing the total DNA content of the samples, as previously described [42,43] using CyQUANT Cell Proliferation Assay kit (Molecular Probes, Thermo Fisher Scientific, Waltham, Massachusetts, USA). Briefly, hBMSCs were lysed with Triton-X 100 (Sigma-Aldrich) and frozen (−70 °C). After two freeze-thaw cycles, 20 μl of cell lysate was mixed with CyQUANT working solution in 96-well plate (Nunc, Thermo Fisher Scientific). The resulting fluorescence was measured at 480/520 nm using a Victor Nivo Multimode Microplate Reader (PerkinElmer, Waltham, Massachusetts, USA).

##### 2.4.1.5 Alkaline phosphatase activity

Alkaline phosphatase (ALP) activity was quantitatively determined (n = 3) on days 1 and 7, as previously described [43]. ALP activity was determined from the same Triton-X 100 cell lysate as the DNA content. Briefly, 20 μl of cell lysate were mixed with a working solution in 96-well plate. The working solution comprised of a 1:1 mixture of 4 mg/ml phosphatase substrate solution (p-nitrophenol phosphate, Sigma-Aldrich) and 1.5 M alkaline buffer solution (2-amino-2-methyl propanol, Sigma-Aldrich). ALP and p-nitrophenol phosphate produce a colorimetric reaction in alkaline environment provided by the buffer solution [38]. The resulting absorbance was measured at 405 nm using the Victor Nivo Multimode Microplate Reader to determine the relative ALP activity in the samples. Finally, the ALP activity for each sample was normalized by the total DNA content.

##### 2.4.1.6 Immunocytochemical staining and confocal microscopy

Immunostaining was performed to evaluate collagen I and osteocalcin expression of the hBMSCs. Phalloidin staining of actin cytoskeleton was performed to assess hBMSC morphology. The stainings were performed as previously described, with slight modifications (Ojansivu et al., 2015). The samples were fixed in 4% paraformaldehyde at RT for 20 min, after which the cells were permeabilized with 0.1 % Triton-X 100 at RT for 15 min. Samples were blocked with 2% bovine serum albumin (BSA) at RT for 1 h, after which the samples were stained overnight with rabbit monoclonal anti-collagen I (1:250 in 1% BSA; Abcam, Cambridge, UK) and mouse monoclonal anti-osteocalcin antibodies (10 μg/ml in 1% BSA; R&D Systems, Bio-Techne, Minneapolis, Minnesota, USA) at 4 °C. The next day, the samples were incubated in a mixture of secondary antibodies (donkey anti-rabbit Alexa Fluor 488 IgG, donkey anti-mouse Alexa Fluor 568 IgG; Invitrogen, Thermo Fisher Scientific) and ATTO 643 phalloidin (ATTO-TEC GmbH, Siegen, Germany; 1:400 in 1% BSA dilution for all) at RT for 1 h. Finally, the cell nuclei were stained with 4′,6-diamidino-2-phenylindole (DAPI, 0.67 μg/ml in DPBS; Sigma-Aldrich) at RT for 30 min.

Samples were imaged using Leica DMi8 Inverted Microscope and Zeiss LSM 780 Confocal Microscope (Zeiss Microscopy, Jena, Germany) with 10× air immersion objective at a resolution of 2048 × 2048 pixels and image size of 1062.7 × 1062.7 μm; z-step size of 1.7 μm was used. Images taken with the inverted microscope were processed using Fiji ImageJ software (version 2.3.0) and the images taken with the confocal microscope using Imaris Microscopy Image Analysis software (version 9.9.1, Oxford Instruments, Abingdon, UK).

##### 2.4.1.7 Quantification of collagen content

Total collagen amount in the samples was determined on days 14 and 21. Scaffolds were transferred to a new 24-well plate and washed with PBS. Collagen was extracted by incubating the scaffolds overnight at 4 °C in 0.5 M acetic acid (Merck, Darmstadt, Germany) with 0.1 mg/ml pepsin (Sigma-Aldrich), with gentle shaking. The assay was then performed using Sircol Soluble Collagen Assay kit (Biocolor, Carrickfergus, UK), following the manufacturer's general protocol and using the provided reagents. At the end of the assay absorbance was measured in duplicate at 544 nm using the Victor Nivo Multimode Microplate Reader.

#### 2.4.2 Human peripheral blood-derived monocyte-differentiated osteoclasts

##### 2.4.2.1 Human monocyte isolation

Monocytes were isolated from buffy coats obtained from healthy donors through the Finnish Red Cross Blood Service (Helsinki, Finland). Buffy coats were prepared from peripheral blood collected under strict regulatory oversight. Peripheral blood mononuclear cells (PBMCs) were first isolated by density gradient centrifugation using Ficoll-Paque PLUS (GE Healthcare, density: 1.077 g/mL) according to the manufacturer's protocol. The PBMC layer was carefully collected and washed three times with phosphate-buffered saline (PBS) to remove platelets and residual Ficoll. Monocytes were then isolated from the PBMC fraction using magnetic-activated cell sorting (MACS). CD14^+^ monocytes were positively selected using CD14 MicroBeads (Miltenyi Biotec, Bergisch Gladbach, Germany) following the manufacturer's instructions.

##### 2.4.2.2 Evaluation of the impact of sintering temperature on osteoclast attachment and bioactivity

For evaluation of the impact of sintering temperature on osteoclast attachment and bioactivity, monocytes were pre-differentiated towards osteoclasts on 6-well plates. Monocytes were plated at a density of 1.5 × 10^5^ cells/cm^2^ in α-MEM containing 10% fetal bovine serum (FBS, Sigma-Aldrich), and 100 U/mL penicillin and 100 μg/mL streptomycin (Euroclone S.p.A., Pero, Italy) further supplemented with 25 ng/mL recombinant human macrophage colony-stimulating factor (M-CSF, R&D systems, Minneapolis, MN, USA), and 25 ng/mL recombinant human receptor activator of nuclear factor κ-B ligand (RANK-L, Gibco, Thermo Fisher Scientific). Medium was changed every 2 or 3 days. After 14 days of culture, mature osteoclasts were detached by incubation for 30 min in Accutase (Sigma-Aldrich) and scraping with a sterile plastic cell scraper.

The sintered HAp-based discs were sterilized and incubated for 24 h in α-MEM containing 10% fetal bovine serum (FBS, Sigma-Aldrich), 100 U/mL penicillin and 100 μg/mL streptomycin. The medium was removed, and mature osteoclasts were added as a single 10 μl droplet of cell suspension containing 5 × 10^4^ cells and incubated for 3 h in an incubator (37 °C, 5% CO_2_). The discs were carefully washed in PBS and placed in a 48-well plate with α-MEM containing 10% FBS, 1 × penicillin-streptomycin, 25 ng/mL M-CSF, and 25 ng/mL RANK-L. Discs without cells were processed similarly and used as controls.

##### 2.4.2.3 Effect of Sr, Zn, Mg-substitutions on osteoclast attachment and bioactivity

HAp2, HAp2_1MIX and HAP2_5MIX-based scaffolds were sterilized and incubated in medium as described for the discs. A total of 2 × 10^5^ freshly isolated human monocytes were seeded onto the scaffolds as four 10 μL droplets of cell suspension. After 90 min of incubation (37 °C, 5% CO_2_), wells were filled with α-MEM containing 10% FBS, 100 U/mL penicillin and 100 μg/mL streptomycin, supplemented with 25 ng/mL M-CSF, and 25 ng/mL RANK-L. Scaffolds were cultured for 14 days with medium changed every 2 or 3 days. Conditioned medium was collected as described for the discs and stored at −80 °C until further analysis.

##### 2.4.2.4 Fluorescent and enzymatic staining for osteoclast visualization

Scaffolds were washed in PBS and fixed in 4% paraformaldehyde at RT for 15 min, permeabilized with 0.1 % Triton-X 100 at RT for 12 min and blocked in 1% BSA for 30 min. Scaffolds were then stained with 1.6 μg/mL TRITC labelled phalloidin (Sigma-Aldrich) for 45 min at RT followed by washes in PBS. Nuclei were stained using 0.5 μg/ml DAPI for 5 min at RT. Samples were imaged using Leica DMi8 Inverted Microscope with 5 × and 20 × objectives. Images were processed with LAS X software (Leica Microsystems). Cells were also stained for osteoclast-specific tartrate resistant acid phosphatase (TRAcP) with an enzymatic stain using the Leucocyte acid phosphatase kit (Sigma-Aldrich) according to the manufacturer's instructions. The staining was imaged with the Nikon Eclipse TE2000 S inverted microscope using a 4 × objective.

##### 2.4.2.5 TRAcP activity assay

TRAcP activity in the conditioned medium was quantified as an indicator of relative osteoclast number on the scaffolds. The assay was performed with slight modifications from a previously described protocol [44]. Briefly, 20 μl of conditioned medium was added to a 96-well plate and incubated with 80 μl of freshly made reaction buffer containing 0.25 M acetic acid (Merck), 0.125% triton x-100 (Sigma-Aldrich), 0.25M NaCl (VWR International, Radnor, PA, USA), 2.5 mM EDTA (Sigma-Aldrich), 6 mM l-ascorbic acid 2-phosphate, 25 mM sodium tartrate dibasic dihydrate (Sigma-Aldrich), and 2.25 mg/ml p-nitrophenol phosphate adjusted to pH 5.5. The reaction mixture was incubated for 1 h at 37 °C and stopped by adding 100 μl of 0.3 M NaOH (Sigma-Aldrich). Absorbance was measured at 405 nm using a Victor NIVO Multimode Microplate Reader (PerkinElmer). Medium from scaffolds without cells were used as a blank control.

##### 2.4.2.6 Scanning electron microscopy

Biomaterials (HAp-based discs and scaffolds) were washed in 0.1 M phosphate buffer (pH 7.4) and fixed in 2.5% glutaraldehyde (Sigma-Aldrich) for 2 h. After washing in phosphate buffer, the materials were dehydrated through an ascending series of ethanol (30%, 50%, 70%, 90%, and 100%, Anora Group Oyj, Helsinki, Finland), with each concentration applied for two intervals of 15 min. This was followed by incubation in 1:2 and 2:1 vol ratio mixtures of hexamethyldisilazane (HMDS, Sigma-Aldrich) and 100% ethanol for 20 min each. Finally, materials were immersed in 100% HMDS and allowed to air dry overnight. The attachment of osteoclast cells was analyzed using a scanning electron microscope (SEM, JSM-IT500LA, Jeol, Japan) at an accelerating voltage of 15 kV. Prior to imaging, discs with osteoclasts were coated with a thin conducting carbon layer.

#### 2.4.3 Inductively coupled plasma optical emission spectrometry

To better understand the biochemical environment of the cells (hBMSCs and osteoclasts) when cultured on the fabricated discs and scaffolds, the culture media was analyzed using inductively coupled plasma optical emission spectrometry (ICP-OES). ICP-OES was performed using 5110 ICP-OES, Agilent Technologies, to quantify the concentration of calcium (*λ* = 397 nm) and phosphorus (*λ* = 215 nm) elements in the conditioned medium. After 3 days of osteoclast culture on the discs, after 14 days of osteoclast culture on the scaffolds, and after 1, 3 and 7 days of hBMSCs culture on non-substituted and substituted scaffolds, conditioned medium was collected and centrifuged for 10 min at 1000 g at 4 °C. Once all samples were collected, they underwent a freeze-thaw cycle, and were diluted in distilled water at 1:10 vol ratio 24 h before the analysis.

### 2.5 Statistical analyses

In the analyses, each group of samples is represented by three replicates. Quantitative results are presented as mean ± standard deviation (SD). Statistical analysis was conducted using one-way analysis of variance (ANOVA), with statistical significance denoted by p < 0.05 (∗) when significant differences were observed.

## 3 Results

### 3.1 Sintering temperature optimization and biomaterial design

#### 3.1.1 Sintering temperature: impact on osteoclast cell attachment and bioactivity

**Fig. 2:**
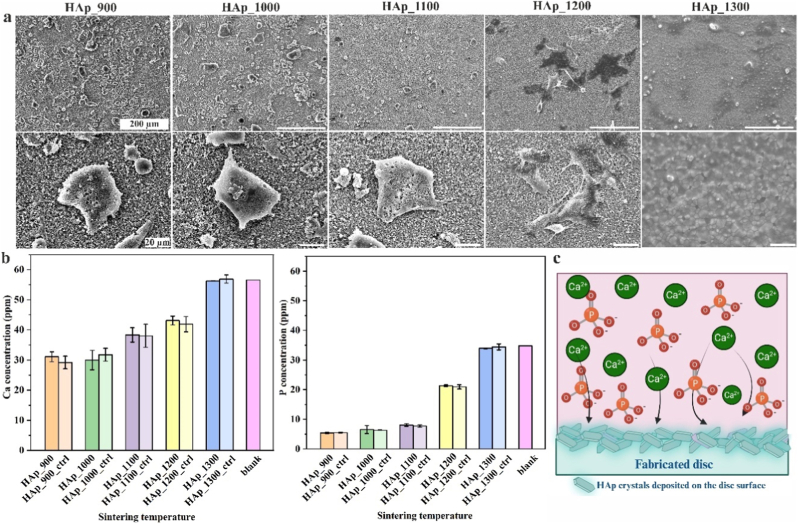
(a) Osteoclast attachment (n = 3, error bars represent SD) on the HAp discs sintered at indicated temperatures observed by SEM. Scale bar: 200 and 20 μm. (b) Calcium (Ca) and phosphorus (P) concentrations (n = 3) in the medium after 3 days of osteoclast culture on HAp discs sintered at different temperatures determined by ICP-MS method. As controls, calcium and phosphorus concentrations were measured in medium where HAp discs were incubated without cells, and in blank medium without HAp discs. (c) Schematic illustration of HAp crystal deposition on the surface of sintered HAp discs. Created with BioRender.com.

To determine the best sintering temperature for cell attachment, HAp-based discs were sintered at 800 °C, 900 °C, 1000 °C, 1100 °C, 1200 °C and 1300 °C. Human osteoclasts were differentiated from monocytes and then detached and seeded onto the discs. Osteoclasts were utilized to determine the best sintering temperature of HAp in selected temperature range, as their morphology is dependent on surface properties and serves as a reliable indicator of surface suitability for cell attachment. Their distribution and morphology were assessed after 3 days of culture (Fig. 2a). Cells were uniformly distributed on fabricated discs (HAp_900, HAp_1000, HAp_1100 and HAp_1200), with the highest cell density observed on discs sintered at 900 °C, while no cells were observed on discs sintered at 1300 °C. Cell number decreased with increasing sintering temperature, indicating negative effect of higher temperatures on cell attachment. In addition, it was observed that cells on discs sintered at 1200 °C exhibited a more elongated morphology and were anchored to the surface via thin, focal adhesion-like structures resembling filopodia. This attachment pattern suggests a reduced adhesion strength compared to osteoclasts observed on samples sintered at lower temperatures. Filopodia, slender, actin-dense extensions from the cell membrane, serve primarily in environmental sensing and initial surface contact. Unlike other cellular regions, filopodia do not contribute significantly to the generation or maintenance of mechanical forces [45,46].

The bioactivity of HAp materials is highly affected by sintering temperatures. To assess ion uptake, material dissolution and osteoclast-mediated resorption, calcium and phosphorus concentrations were measured from the conditioned medium after 3 days of osteoclast culture on all fabricated discs (Fig. 2b). Control measurements were performed using medium incubated with HAp discs in the absence of osteoclasts, as well as medium without any disc incubation. No significant difference in calcium and phosphorus concentrations were observed between discs incubated with and without osteoclasts, indicating that ion exchange is mainly attributed to material characteristics rather than osteoclastic resorption. However, significant difference in calcium and phosphorus ion uptake was observed between discs sintered at different temperatures. The highest ion uptake occurred on discs sintered at 900 °C, 1000 °C, and 1100 °C, followed by a moderate uptake on discs sintered at 1200 °C. In contrast, no ion uptake was detected for 1300 °C compared to the blank control. These results suggest that at lower sintering temperatures, the surface of the HAp discs remains sufficiently reactive to support the formation of a new apatite phase, as schematically illustrated in Fig. 2c, indicating good bioactivity. Based on these findings, a sintering temperature of 1000 °C was selected for further studies on porous HAp scaffolds, as it provided favourable cell attachment and bioactivity. Although lower temperatures such as 900 °C showed slightly enhanced cell attachment, sintering temperature of 1000 °C was preferred to ensure sufficient mechanical properties as a result of enhanced biomaterial densification.

#### 3.1.2 Effect of different porosities on microstructure and mechanical properties

**Fig. 3:**
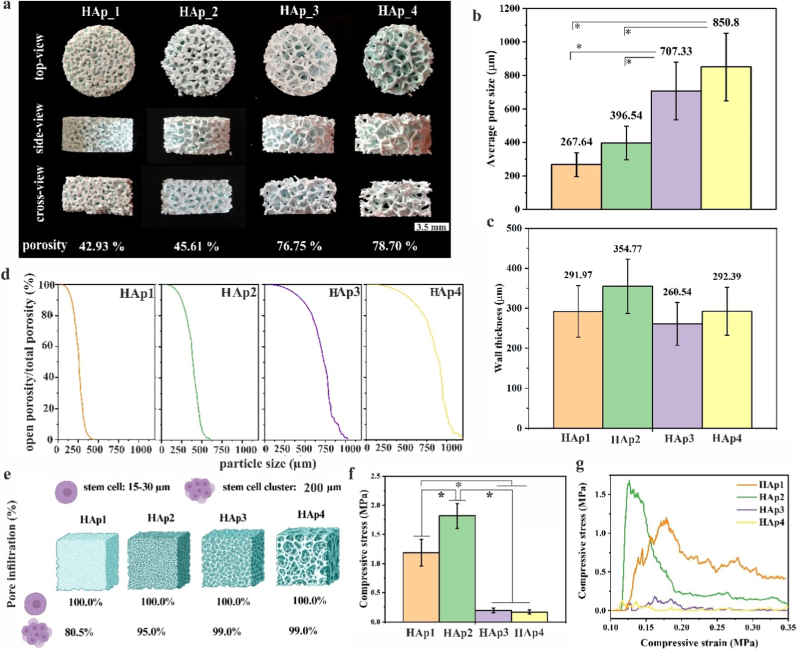
(a) Morphological analysis (n = 1, error bars represent SD) of four different scaffolds (HAp1, HAp2, HAp3, and HAp4) from top, side, and cross-sectional views observed by digital camera, along with porosity evaluation values determined by micro-CT. Scale bar: 3.5 mm. Average (b) pore size and (c) wall thickness of fabricated scaffolds determined by micro-CT. (d) The relationship between open porosity/total porosity (%) and particle size, as determined through the analysis of micro-CT images of the scaffolds. (e) Schematic illustration and percentages on how many pores can be infiltrated by particles of 30 (stem cells) and 200 μm (stem cells clusters) in size according to a relationship between open porosity/total porosity (%) and particle size. Created in https://BioRender.com (f) Compressive strength (n = 7, error bars represent SD) and (g) representative experimental stress-strain response determined by compression test. Source data are available at: https://doi.org/10.5281/zenodo.17286078. The significant difference between two groups (∗): p < 0.01.

Scaffolds with four different microstructures HAp1, HAp2, HAp3 and HAp4 based on pure HAp phase were fabricated via ceramic VPP and sintered at 1000 °C (Fig. 3a). Key internal parameters of scaffolds for bone regeneration were determined by micro-CT analysis and include: (a) total porosity (Fig. 3a) that define number of total void spaces, affecting mechanical properties, (b) average pore size (Fig. 3b) affecting bone tissue ingrowth, (c) wall thickness (Fig. 3c) affecting cell penetration and tissue ingrowth, and (d) proportion of open porosity compared to the overall porosity as a function of certain sized particles (Fig. 3d) that describes level of pore interconnectivity and provides number (%) of pores that are accessible to particles of a certain size, while of particular interest in present study is size of used stem cells [42,47]. The morphological analysis showed highly porous scaffold structures, while cross-sectional views confirmed the effectiveness of the cleaning process as all pores appeared to be open without evidence of clogged pores. The average wall thickness was similar across all scaffold types, with HAp2 showing a slightly higher value without statistical significance. Open porosity describes the interconnectivity of pores, a continuous network that supports the penetration and distribution of cells or particles seeded on scaffold. The size of stem cells is in the range of 15–30 μm [48]; however, particle sizes up to 200 μm are highly relevant when evaluating cell seeding efficiency as cells tend to form small aggregates. Dense cell aggregates have demonstrated superior tissue formation and cell viability compared to individual cells [42]. As shown in Fig. 3d–e, 30 μm particles are able to infiltrate into all pores in fabricated scaffolds, while 200 μm particles are able to infiltrate into 80.5 % of the pores in scaffold HAp1, 95.0 % of the pores in scaffold HAp_2, and 99.0 % of the pores in scaffold HAp3 and HAp4.

To determine the effect of different microstructures designs of scaffolds on mechanical properties, compressive strength of the porous scaffolds was assessed in terms of the measured fracture stress (Fig. 3f). As expected, scaffolds HAp3 and HAp4 exhibited significantly lower compressive strength compared to HAp1 and HAp2, which can be attributed to the scaffolds' higher porosity and larger pore sizes. Scaffolds HAp2 demonstrated higher compressive strength than HAp1. This unexpected result can be explained by the greater wall thickness observed in HAp2 compared to HAp1, HAp3 and HAp4 scaffolds, as confirmed by microstructural analysis using micro-CT measurements. The representative stress-strain curves of compression test are shown in Fig. 3g. Fabricated scaffolds with different microstructure designs exhibited characteristic stress–strain behavior typical of complex porous biological biomaterials. This response is characterized by a repeated loss and recovery of strength as layers of the scaffold walls collapse under compression. Initially, elastic stress increases linearly. With further compression, the resulting stress–strain profile shows a sawtooth-like behaviour. When the stress within a wall exceeds the characteristic fracture stress of the material, the wall fails. The wall failure does not lead to a complete structural collapse but results in a release of overall elastic stress, quickly recovering as displacement increases. The curve profile is related to the structural design and interactions within the scaffold, such as the arrangement, failure, or deformation of walls and pores [49,50].

### 3.2 Biological characterization of designed HAp-based scaffolds

#### 3.2.1 Role of scaffold microstructure in modulating osteogenic responses

Stem cells have the potential to differentiate into different cell types depending on the environment, including matrix-producing osteoblasts. The process of osteogenic differentiation can be divided into three stages: (i) proliferation, (ii) formation of ECM and cell maturation, and (iii) ECM mineralization. Each stage is characterized by expressions of characteristic differentiation markers [51]. Therefore, in the present study, hBMSCs were seeded on the scaffolds with distinct designs, to determine which microstructure has the highest osteogenic potential.

##### 3.2.1.1 Cell proliferation and ALP activity

**Fig. 4:**
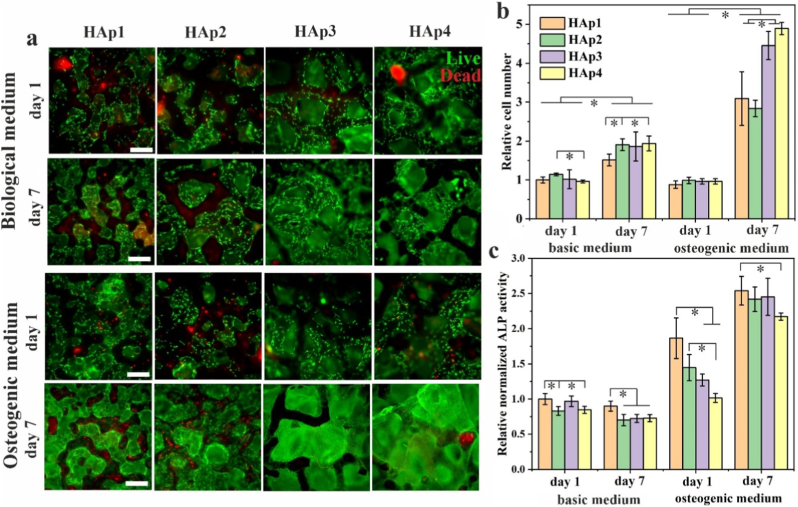
Biological characterization of HAp1, HAp2, HAp3 and HAp4 scaffolds. (a) Qualitative (n = 2) and (b) quantitative (n = 3, error bars represent SD) analysis of seeded hBMSC after 1 and 7 days of cell culture obtained by *live/dead* and CyQUANT assay, respectively. Live cells are stained green; dead cells are stained red. (c) ALP activity (n = 3, error bars represent SD) normalized to the cell amount. Scale bar: 500 μm. Source data are available at: https://doi.org/10.5281/zenodo.18506207. The significant difference between two groups (∗): p < 0.01. (For interpretation of the references to color in this figure legend, the reader is referred to the Web version of this article.)

The *live/dead* assay showed homogeneously dispersed hBMSCs with good viability and cell–cell interactions after 1 and 7 days of cell culture for all scaffolds cultured in basic medium (BM) and osteogenic medium (OM) (Fig. 4a). The increase in number after 7 days of cell culture is evident for all scaffold types when osteogenic medium was used, while slight increase in cell viability after 7 days of cell culture can be observed when BM was used. Dead cells were observed on the upper surface walls of the scaffolds, which were in direct contact with the culture plate where cells did not have direct access to the medium. However, no dead cells were detected within the internal regions of the scaffolds. In addition, after 7 days of cell culture in osteogenic medium cells started to form confluent layers in scaffolds HAp3 and HAp4. Quantitative analysis of cell number (Fig. 4b) aligned with the *live/dead* assay results, showing a significant increase of cell viability after 7 days of cell culture in both BM and OM. Higher increase in cell viability was evident after 7 days when OM was used. A significant difference was observed between samples HAp2 and HAp4, where after day 1 in BM there is significantly more cells viable on scaffold HAp2. Further, after 7 days of cell culture in OM, viable cells are significantly higher in number in samples HAp3 and HAp4, compared to samples HAp1 and HAp2. This can be explained by the larger pore size and higher porosity in scaffolds HAp3 and HAp4, which provides more space for cell growth, and enhance the diffusion of nutrients and oxygen.

In addition to assessing cell viability, it is important to analyse the expression of osteogenic differentiation markers [52]. Results of ALP activity normalized to the cell amount is shown in Fig. 4c. Significantly lower ALP activity was observed in scaffolds cultured in BM compared to when OM was used. After 7 days of cell culture in BM, ALP activity in HAp1 scaffolds was significantly higher compared to scaffolds HAp2, HAp3 and HAp4. When cultured in OM, ALP activity decreased with increasing pore size and porosity at day 1. This difference was statistically significant between HAp1 scaffolds and both the HAp3 and HAp4 scaffolds. The difference was less pronounced after 7 days of culture in OM, although ALP activity in HAp1 was still significantly higher when compared to scaffold HAp4.

##### 3.2.1.2 Immunocytochemical staining and quantification of collagen expression

**Fig. 5:**
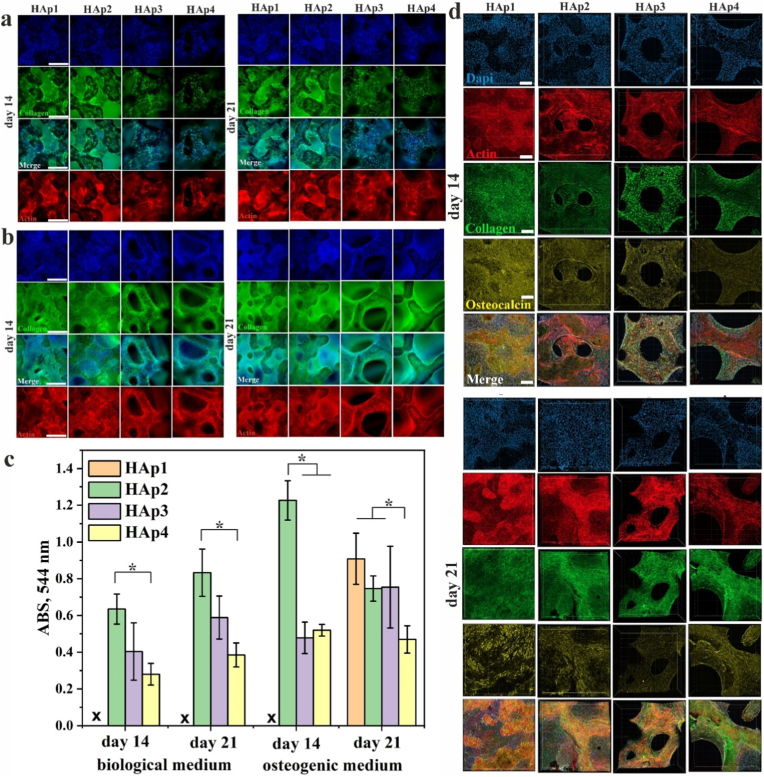
The expression of collagen type I (n = 3, error bars represent SD) on the scaffolds seeded with hBMSCs after 14 and 21 days of cell culture in (a) basic and (b) osteogenic medium determined by immunocytochemical staining. Cell nuclei stained with DAPI (blue), actin cytoskeleton stained with phalloidin (red) and an antibody for human Coll I (green). Scale bar: 500 μm. (c) Quantitative analysis of total collagen content after 14 and 21 days of cell culture in basic medium and osteogenic medium. The significant difference between two groups (∗): p < 0.01. (d) The expression of Coll I and OCN on the scaffolds seeded with hBMSCs after 14 and 21 days of cell culture in osteogenic medium determined by immunocytochemical staining. Cell nuclei stained with DAPI (blue), actin cytoskeleton stained with phalloidin (red), an antibody for human Coll I (green), and an antibody for human OCN (yellow). Scale bar: 100 μm. (For interpretation of the references to color in this figure legend, the reader is referred to the Web version of this article.)

A qualitative evaluation of osteogenic differentiation in hBMSCs cultured in BM on HAp1, HAp2, HAp3 and HAp4 scaffolds was examined using widefield fluorescence microscopy (Fig. 5a). Collagen type I (Coll I) is the main organic component of the bone ECM and therefore was selected as a differentiation marker. Immunohistochemical analysis revealed Coll I secretion after 14 and 21 days of cell culture in BM, signifying the good osteogenic potential of all scaffold groups. More Coll I was observed in scaffolds HAp1 and HA2, where fibril-like collagen network was secreted outside of the cells implying ECM formation instead of mainly being expressed intracellularly as evident in scaffolds HAp3 and HAp4. Phalloidin staining of the actin cytoskeleton was performed alongside immunocytochemical staining for Coll I and nuclear staining to assess cellular organization and interactions. All scaffold types exhibited a highly interconnected actin network, suggesting strong cell–cell interactions essential for bone regeneration. After 14 and 21 days of culture, scaffolds HAp1 and HAp2 shown well-organized, linear actin filaments, indicating organized cytoskeletal structures, enhanced cellular organization and favourable cell attachment.

The expression of Coll I after 14 and 21 days of cell culture in OM (Fig. 5b) further showed the effect of different scaffold microstructure designs on Coll I deposition and cellular organization. Compared to the results obtained in BM, both Coll I expression, cell number and cell–cell interactions were significantly enhanced in OM. After 14 days, fibrillar Coll I expression was particularly evident in scaffolds HAp1 and HAp2, with Coll I localized not only intracellularly but also in the extracellular space. Furthermore, staining for nuclei, actin, and Coll I revealed cellular presence within the HAp1 and HAp2 scaffold pores, and not only on the scaffold walls, as evident for HAp3 and HAp4 scaffolds. This suggests that cells had begun to infiltrate the pore voids and initiate ECM production. A similar effect was observed after 21 days of cell culture, strengthening the conclusion that the porosity and pore size distribution of HAp1 and HAp2 scaffolds support good cell–cell interactions and promote conditions for efficient bone tissue regeneration. Results of quantitative analysis of total collagen content after 14 and 21 days of cell culture in BM and OM are shown in Fig. 5c. We were not able to quantify collagen in scaffolds HAp1, except for scaffolds cultured for 21 days in OM. Collagen content decreased with increasing pore size and porosity, with significantly higher secretion observed for scaffold HAp2 compared to scaffold HAp4 at both time points. Further, total collagen content was significantly higher in scaffold HAp2 after 14 days of culture in OM compared to scaffold HAp3 and HAp4. After 21 days of culture in OM, collagen content was similar in scaffolds HAp1, HAp2 and HAp3. However, total collagen content was significantly lower in HAp4 scaffolds compared to scaffolds HAp1 and HAp2.

##### 3.2.1.3 Immunocytochemical staining: expression of collagen and osteocalcin

To further confirm the osteogenic potential of scaffolds and determine which scaffold design is most promising for bone regeneration, expression of osteocalcin (OCN), a late marker of osteoblast differentiation, was studied alongside with Coll I expression. Fig. 5d presents the immunocytochemical evaluation of middle-stage osteogenic marker Coll I and late-stage osteogenic marker OCN, produced by BMSCs cultured in OM in scaffolds HAp1, HAp2, HAp3, and HAp4. Cell density was similar in all scaffolds at both time points with positive expression of both Coll I and OCN. After 14 days of cell culture, Coll I and OCN expression was intracellular on scaffolds HAp3 and HAp4, while extracellular production was evident on scaffolds HAp1 and HAp2. BMSCs were present within the pores of scaffolds HAp1 at 14 days of culture. After 21 days of cell culture, Coll I and OCN secretion was detected in all scaffolds. OCN expression was higher within scaffolds HAp1 and HAp2. BMSCs expressing Coll I and OCN filled the pores of scaffolds HAp1 and HAp2, whereas the pores of scaffolds HAp3 and HAp4 remained devoid of cells and matrix.

### 3.3 Physiochemical and biological properties of Sr, Zn, Mg-substituted scaffolds

#### 3.3.1 Effect of Sr, Zn, Mg-substitutions on physiochemical properties

**Fig. 6:**
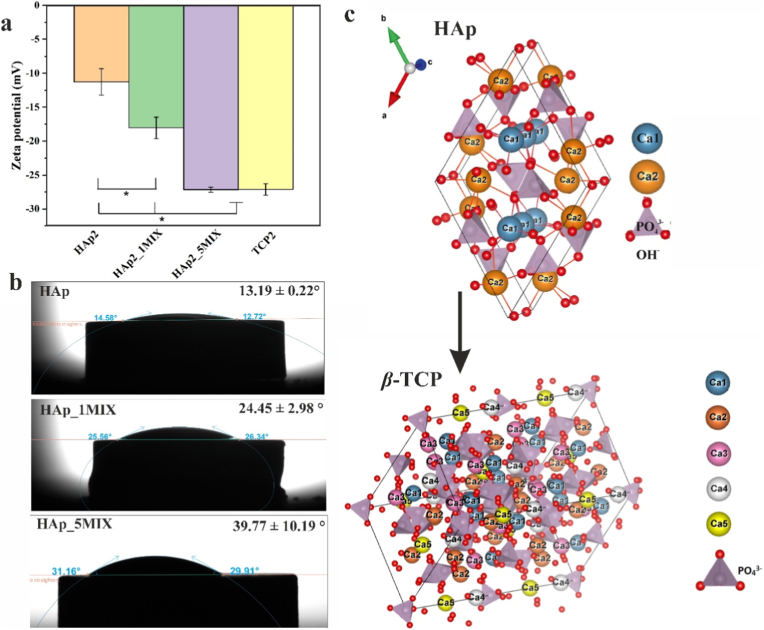
(a) ζ-potential measurements (n = 1, error bars represent SD, 3 cycles) for scaffolds HAp2, HAp2_1MIX, and HAp2_5MIX. The significant difference between two groups (∗): p < 0.01. Source data are available at: https://doi.org/10.5281/zenodo.17286078. (b) Representative images and average water contact angle (°) measurements (n = 3) for HAp, HAp_1MIX and HAp_5MIX discs sintered at 1000 °C. Source data are available at: https://doi.org/10.5281/zenodo.18504934 (c) Schematic illustration of HAp crystal lattice that transforms to *β*-TCP crystal lattice obtained by software VESTA 3 [53].

Detailed SEM, energy-dispersive *X*-ray spectroscopy, *X*-ray diffraction, and mechanical characterizations of the HAp2, HAp2_1MIX, and HAp2_5MIX scaffolds have been reported in our previous study [20]. To further explore physiochemical properties of designed scaffolds, ζ-potential (Fig. 6a) analysis was performed along with wettability test by contact angle measurements (Fig. 6b). In our previous study, mineralogical phase composition of HAp2, HAp2_1MIX and HAp2_5MIX scaffolds was determined [20]. *X*-ray diffraction patterns of the scaffold HAp2 closely matched the line pattern for HAp (ICDD No. 09–0431). However, in the case of HAp2_1MIX and HAp2_5MIX scaffolds, additional peaks corresponding to *β*-TCP (ICDD No. 09–0169) were observed, with higher peak intensity in the powder containing a higher substitution level of Sr^2+,^ Mg^2+^, and Zn^2+^ ions (Fig. 6c). Therefore, a scaffold based on *β*-TCP was used as a control for ζ-potential measurements. The results revealed that all samples were negatively charged. The ζ-potential of the scaffolds decreased with increasing substitution levels of Sr^2+^, Mg^2+^, and Zn^2+^ ions. The ζ-potential of *β*-TCP scaffolds was similar to the one characterizing HAp2_5MIX scaffolds, indicating that the decrease in ζ-potential with increasing substitution levels was related to the increasing content of the *β*-TCP mineralogical phase. The wettability of HAp, HAp_1MIX, and HAp_5MIX was evaluated using contact angle measurements on discs sintered at 1000 °C. The average contact angle values were 13.19 ± 0.22° for HAp, 24.45 ± 2.98° for HAp_1MIX, and 39.77 ± 10.19° for HAp_5MIX, indicating an increase in contact angle with increasing substitution levels and change in mineralogical composition.

#### 3.3.2 Effect of Sr, Zn, Mg-substitutions on biological properties

**Fig. 7:**
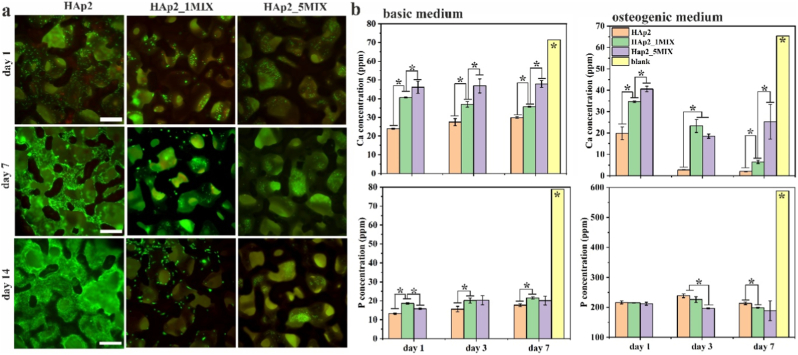
(a) Qualitative (n = 2) analysis of seeded hBMSC after 1, 7 and 14 days of cell culture obtained by *live/dead* and assay on scaffolds HAp2, HAp2_1MIX, and HAp2_5MIX. Live cells are stained in green; dead cells are stained in red. (b) Calcium (Ca) and phosphorus (P) concentrations (n = 3, error bars represent SD) in the medium after 1, 3 and 14 days of hBMSC culture on HAp2, HAp2_1MIX, and HAp2_5MIX scaffolds in osteogenic (OM) and biological (BM) medium determined by ICP-MS analysis. As control, calcium and phosphorus concentrations in biological and osteogenic mediums were used. The significant difference between two groups (∗): *p* < 0.01. (For interpretation of the references to color in this figure legend, the reader is referred to the Web version of this article.)

To examine the effect of ion substitutions on the biological performance of designed biomaterials, the HAp2 scaffold was selected for further studies due to its demonstrated mechanical and osteogenic properties. A *live/dead* assay was performed on hBMSCs on scaffolds HAp2, HAp2_1MIX and HAp2_5MIX at 1, 7, and 14 days of culture in both BM and OM. Only results in OM are shown (Fig. 7a) as cultures in BM showed similar results as in OM (data not shown). BMSCs were homogeneously dispersed on HAp2 scaffolds, with good viability and evident cell–cell interactions, along with a noticeable increase in cell number over time. Scaffolds HAp2_1MIX, containing 1 mol% of substitution with Sr^2+^, Mg^2+^, and Zn^2+^, contained less cells. Least cells were observed on scaffolds HAp2_5MIX, containing 5 mol% of substitution with Sr^2+^, Mg^2+^, and Zn^2+^, with cells located in inner parts of scaffold with round cell morphology. Calcium and phosphorus concentration were determined at 1, 3 and 7 days of culture for all examined scaffolds as shown in Fig. 7b. Compared to culture medium from an empty well (blank), significant differences in calcium ion uptake was observed for all prepared scaffold in both BM and OM. The ion uptake follows the order as HAp2 > HAp2_1MIX > HAp2_5MIX with significant differences between scaffolds. Significant ion uptake compared to blank was observed for phosphorus ion concentration. In OM, however, differences between scaffolds were small and only statistically significant at 3 and 7 days.

**Fig. 8:**
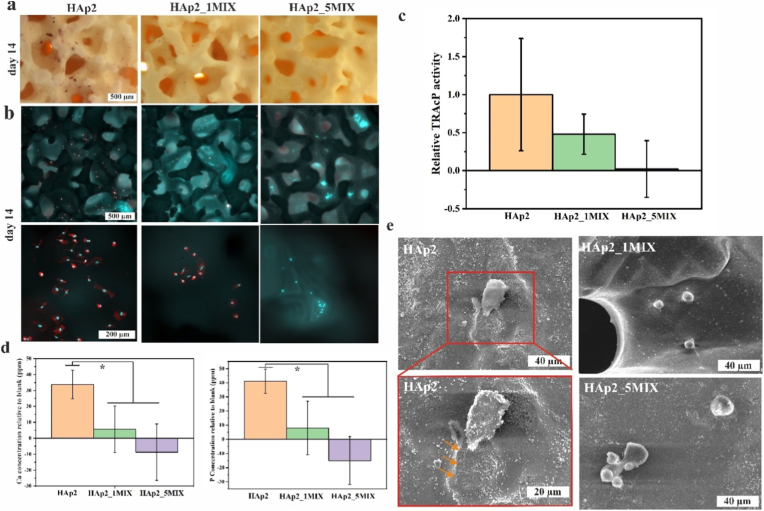
Osteoclast formation (n = 3, error bars represent SD) on HAp2, HAp2_1MIX, and HAp2_5MIX scaffolds after 14 days of cell culture characterized by (a) enzymatic staining of osteoclast-specific TRAcP (red), (b) fluorescent staining where cell nuclei were stained with DAPI (blue) and actin cytoskeleton was stained with phalloidin (red), and (c) TRAcP activity assay. Scale bar: 500 and 200 μm. (d) Calcium and phosphorus concentrations determined by ICP-MS analysis in the medium after 14 days of osteoclast culture on HAp2, HAp2_1MIX, and HAp2_5MIX scaffolds relative to a scaffold without osteoclasts. The significant difference between two groups (∗): *p* < 0.01. Source data are available at: https://doi.org/10.5281/zenodo.18592493 (e) HAp2, HAp2_1MIX, and HAp2_5MIX scaffolds microstructure determined by SEM after 14 days of osteoclast cell culture. Resorption trail indicated by orange arrows. Scale bar: 40 and 20 μm. (For interpretation of the references to color in this figure legend, the reader is referred to the Web version of this article.)

To further examine the cell behaviour, osteoclast formation was examined on the scaffolds HAp2, HAp2_1MIX and HAp2_5MIX. Monocytes were seeded on the scaffolds and differentiated towards osteoclasts *in situ*. A similar effect was observed for osteoclasts as for hBMSCs. Osteoclast formation was observed for scaffold HAp2 evident by all performed characterization methods, enzymatic staining of osteoclast-specific TRAcP (Fig. 8a), fluorescent staining for actin and cell nuclei (Fig. 8b), and colorimetric quantification of TRAcP activity (Fig. 8c). Less osteoclasts were observed on HAp2_1MIX compared to HAp2 scaffolds, and none were observed on HAp2_5MIX scaffolds. Calcium and phosphorus concentrations were measured using ICP-OES in the conditioned medium at the end of the culture and compared to medium (blank) from an empty culture well (Fig. 8d). The resorption effect of osteoclasts was detected by increased release of calcium and phosphorus on scaffold HAp2, significantly lower ion release on scaffold HAp_1MIX, while ion uptake was visible for scaffold HAp2_5MIX, as no osteoclast cells were active on this scaffold, leading to no released ions due to resorption. SEM analysis (Fig. 8e) of the scaffold surfaces revealed osteoclastic resorption trails on the scaffold HAp2, while no resorption trails were observed on the HAp2_1MIX and HAp2_5MIX scaffolds. Osteoclast on the HAp2_1MIX and HAp2_5MIX scaffolds were round-shaped and smaller in size indicating not-matured osteoclast cells, whereas cells detected on HAp2 exhibited osteoclasts’ characteristic shape and size.

## 4 Discussion

Aim of present study is to (i) evaluate the suitability of ceramic VPP for fabricating biomimetic scaffolds, (ii) determine how scaffold surface and microstructural characteristics influence cellular responses, (iii) identify the most favourable scaffold design among the tested variations, and (iv) assess the biological effects of incorporating Sr, Mg, and Zn ions into the HAp lattice on biomaterials properties and cellular response. Obtained results provide an understanding of how processing parameters, microstructure, and ion incorporation collectively affect the biological performance of VPP-fabricated HAp-based scaffolds, and indicate which research questions should be addressed in future studies.

Ceramic VPP is a novel technique that enables fabrication of highly complex structures and opens possibilities for mass-production of personalized scaffolds [54]. Processing of fabricated materials at high temperatures is required to remove the organic components of the slurry and sinter the ceramic material. Applying high temperatures during biomaterial sintering ensures adequate mechanical characteristics but leads to reduced bioactivity and bioresorption [55]. The advantages and disadvantages of the ceramic VPP fabrication method need to be balanced, and developed biomaterials need to be examined in detail to determine future directions and possibilities for scaffolds fabrication by this method. To effectively guide future scaffold development using ceramic VPP, it is essential to consider biological principles such as bone remodelling, a crucial step in the bone regeneration process. In natural bone tissue, remodelling is a continuous process in which old bone is resorbed by osteoclasts and subsequently replaced with new bone formed by osteoblasts [56]. Osteoclasts, multinucleated cells derived from monocyte-lineage precursors, play a central role in regulating bone metabolism, with osteoclastogenesis serving as the initiating step of the bone remodelling cycle [40,56]. The right balance between the activities of osteoclasts and osteoblasts is required for proper function in natural bone tissue. Similar to the process in natural bone tissue, scaffolds have to be resorbed over time and replaced by new bone. For that reason, understanding osteoblast/osteoclast activity is necessary for a successful biomaterial development [57]. A limited number of studies explore osteoclast behavior: however, studies on resorption properties indicate that the key to designing an osteoinductive CaP-based biomaterials is to first design a biomaterial surface that promotes the function of osteoclasts [58]. Obtained results during osteoclast culture are coherent with the explanation given by Chen et al. [59]: ion release from the biomaterial occurs during the first day of immersion, followed by rapid deposition (uptake) as observed for HAp_900, HAp_1000, HAp_1100 and HAp_1200. No measurable osteoclast resorption activity after 3 days of cell culture might be due to the short time of incubation and/or due to high sintering temperatures slowing down the resorption process [55,58].

The porosity and pore size of scaffolds directly influence their function and application in biomedical applications for bone regeneration. Porosity, pore size and interconnectivity are the basic requirements for tissue engineering scaffolds to accurately simulate the bone matrix and to provide a suitable microenvironment for bone-cell adhesion and proliferation, which guides and promotes new tissue formation [24]. The ceramic VPP method's capability to fabricate highly complex scaffold designs was used to produce biomimetic scaffolds with advanced pore microstructure (irregular pore shapes), which demonstrated superior bone formation capabilities compared to scaffolds with regular pore shapes [60,61]. Wang et al. [60] determined that scaffolds featuring an irregular porous structure enhanced cell behaviour, including adhesion, proliferation, differentiation, bone formation, ingrowth, and vascularization, in contrast to scaffolds with regular pore shapes. Supporting these findings, a study by Liang et al. [61] showed that scaffolds with irregularly shaped pores created a diverse mechanical stimulation environment, including micro-scale deformations, fluid shear stress, and enhanced diffusion of biological factors. This complex environment closely aligned with the requirements for osteogenic mechanical stimulation, demonstrating superior osteogenic properties compared to scaffolds with regularly shaped pores. Both studies indicate the importance of mimicking the complex microstructure of natural bone tissue, highlighting the advantages of irregularly structured scaffolds over those with uniform pore characteristics. However, mimicking bone hierarchical structure on different scale levels remains a significant challenge, and the debate on appropriate scaffold microstructure design for the best regenerative properties is still ongoing [54].

According to Feng et al. [62] an average pore size of at least 300 μm is preferable for promoting new bone formation, while a pore size of 400 μm is considered optimal for vascularization during osteogenesis. An *in vivo* study by Zhao et al. [63] showed that a scaffold with a smaller pore size in the range of 200 – 300 μm showed the best results for ALP activity, histological assessment, and mechanical properties in the early stage of bone formation. Wu et al. [64] fabricated CaP-based scaffolds substituted with magnesium by the ceramic VPP method with different pore struts and pore heights. *In vivo* analyses indicated that bone tissue ingrowth was retarded in ∼200 μm pore size scaffolds, while the scaffolds with pores of 320 μm showed good bone tissue growth within the whole volume of the scaffold, while the neo-tissue ingrowth was observed in the scaffolds with pores of 450 μm and 600 μm. The scaffold with pores of 450 μm showed a higher amount of new bone tissue after 16 weeks *in vivo*, in comparison with the scaffolds containing pores of 600 μm. It was assumed that the faster biodegradation rate in 600 μm scaffolds led to larger pore dimension and a low-density substrate with time, leading to non-optimal conditions for osteogenic cell migration and growth [64]. In the present study, when designing CAD models of scaffolds with different microstructures, it was considered that interconnectivity and a wide pore distribution range are essential for the bone regeneration process. Interconnectivity of pores is a key factor for efficient diffusion of essential nutrients, oxygen, and extracellular fluids into and out of the cellular matrix placed within a porous biomaterial, while different pore sizes play distinct roles in the bone formation process. Pores in the range of 100 – 400 μm enhance osteogenesis by promoting cell migration, formation of cell-cell networks, vascularization, nutrient supply, and the diffusion of metabolic waste, while pores <100 μm are essential for cell seeding and retention, angiogenesis, and interactions between the cells and the ECM [65]. Both small and large pores play different roles in vascularization, and scaffolds having a wide pore size distribution may be the best solution for effective blood vessels formation [66,67]. Results of discussed and present study are not in agreement with studies by Wang et al. [68] and Zhang et al. [69] indicating that material type significantly influences osteogenic performance of designed scaffolds. The study by Wang et al. [68] on tantalum-based scaffolds showed that an average pore size of 500 μm, a pore distribution range of 200–1200 μm, and a porosity of 70 % are optimal for bone repair *in vitro* and *in vivo*. Further, the study by Zhang et al. [69] on a porous cubic cell titanium-based scaffold with cubic cell structure showed that the optimal pore size for enhanced osteogenic response and bone formation was 600 – 700 μm, while scaffolds with smaller or larger pore size negatively affected cellular behaviour and bone regeneration. While various studies on scaffold microstructure design offer valuable guidance for developing new scaffolds, it is important to recognize that the biological response is closely influenced by factors such as microstructure, pore shape, interconnectivity, and the type of biomaterial used. Therefore, the optimal scaffold design should be specifically evaluated for each newly developed biomaterial.

Obtained CAD models in present study were obtained to mimic native trabecular bone tissue. Trabecular bone has a lattice-like architecture with 50%–90% porosity, pore size of 300–500 μm and trabeculae (wall thickness) of 50–1000 μm thick, forming interconnected plates and struts, with marrow filling the intervening spaces. The key geometrical parameters of bone tissue engineering scaffolds (porosity, pore size, pore shape, interconnectivity, and curvature) play a pivotal role in their biological performance. However, the interplay between these parameters and scaffold efficacy remains insufficiently understood [[70], [71], [72], [73], [74]]. In the present study, scaffolds HAp1 with an average pore size of 270 ± 72 μm and HAp2 with an average pore size of ∼400 ± 100 μm showed the best *in vitro* osteogenic response (ALP, Coll I and OCN expression). The ALP is considered an early indicator of cellular response and is the first functional gene expressed in the process of osteogenesis. The levels of ALP activity by cells increase with the progress of osteoblastic differentiation in the early stage and decrease as mineralization has started [52,75,76]. Further, Coll I fibres are the template for mineralization *in vivo* and make an important contribution to bone regeneration during the bone remodelling process [77]. Formation of a Coll I network is the first step towards mineralization, as mature ECM containing Coll I serves as a substrate for CaP growth and can allow spontaneous mineral formation without the necessity for cellular functions [78]. OCN is a non-collagenous protein that contributes to the formation of HAp crystal and bone mineralization and is used as a specific marker of osteoblast differentiation at the final stage [79]. According to the obtained results, HAp1 and HAp2 scaffolds had appropriate microstructure design, containing both small and large pore sizes to ensure efficient nutrient transport, allowing cell penetration into the scaffold to form new bony tissue, and to allow cell-cell interactions and differentiation process. However, scaffold HAp2 (1.70 ± 0.27 MPa) demonstrated higher compressive strength compared to HAp1 (1.22 ± 0.23 MPa). Due to better mechanical performance, scaffold HAp2 was selected for further studies on scaffolds substituted with Mg^2+^, Zn^2+^ and Sr^2+^ ions. Cortical bone has Young׳s modulus in the range 1–20 GPa and a compressive strength in range of 1–100 MPa, while trabecular bone has Young׳s modulus in range 0.1–1.0 GPa and a compressive strength in range 1–10 MPa. Despite significant variability in the reported mechanical characteristics of bone, these values serve as a valuable reference for establishing the necessary mechanical properties for a scaffold [80]. The compressive strength of the fabricated scaffolds falls within the lower range of values reported for trabecular bone, and therefore these scaffolds are not suitable for load-bearing applications. The reduced mechanical performance is primarily attributed to the lower sintering temperature (1000 °C) selected in this study to preserve the bioactivity of HAp and to maintain appropriate cell attachment. In contrast, sintering at the more commonly used temperature of 1300 °C results in significantly improved densification and mechanical strength [20], but as demonstrated in the present work, leads to a loss of bioactivity and poor cell attachment. The findings clearly demonstrate that increased densification and mechanical strength observed in our previous study [20] simultaneously lower bioactivity, making it necessary to balance these two essential properties during scaffold design. Furthermore, natural bone is a hierarchical composite consisting mainly of HAp and collagen type I, which together provide superior mechanical properties. In comparison, scaffolds composed solely of ceramic phases exhibit brittle behavior and consequently lower mechanical strength, especially when sintered at lower temperatures. Future development of VPP should be focused on fabricating composite scaffolds combining bioceramics with polymers to avoid sintering process and to better match the composition and mechanical properties of natural bone tissue.

Synthetic HAp is considered an ideal candidate for obtaining scaffolds for bone tissue regeneration due to its similarity to bioapatite, the inorganic phase of bone tissue. Due to its highly substituted structure, bioapatite with a Ca/P molar ratio less than 1.67, is called calcium-deficient carbonated HAp [81]. The flexible hexagonal structure of HAp can incorporate a variety of ions and mimic the chemical composition of natural bone tissue to further improve the properties of fabricated scaffolds [82]. In the study by Hu et al. [83] the positive effect of Zn^2+^ and Sr^2+^ incorporated on natural bone ceramic on bone repair was confirmed compared to scaffold without incorporated trace element. Obtained scaffolds supported osteogenic differentiation of hBMSCs with simultaneously exhibiting antibacterial effect. In the present study, the positive effect of trace elements was not observed due to changes in mineralogical phases after sintering, suggesting that different strategies of ion incorporation onto fabricated scaffolds need to be explored. However, compared to using natural bone ceramics as template, techniques such as ceramic VPP open new horizons in personalized medicine, where scaffolds can be produced according to the patient's defect, which cannot be achieved by other preparation procedures that do not involve advanced 3D fabrication techniques. Hu et al. [83] results align with our earlier study [25], where composite scaffolds based on biopolymer chitosan and 30 wt% of HAp/octacalcium phosphate pentahydrate, substituted with 1 and 5 mol% Mg^2+^, selenite (SeO_3_^2−^), Sr^2+^ and Zn^2+^ ions, were prepared by the freeze gelation method. Trace elements significantly enhanced osteogenic properties of prepared scaffolds, as evidenced by the increased bone tissue formation and expression of osteogenic marker genes in cultures with hBMSC. Substitution of trace elements led to the formation of calcium deficient HAp that is not stable at higher temperatures but transforms to a more stable CaP phase at these conditions, i.e. *β*-TCP. Stoichiometric HAp, free of ionic impurities, exhibits stability up to 1400 °C, while at higher temperatures transition to *β*-TCP begins [84]. The increased amount of *β*-TCP is a common occurrence during the thermal treatment of non-stoichiometric, calcium-deficient HAp at temperatures exceeding 700 °C (Fig. 6c) [85]. As reported in our previous study [86], the quantitative phase analysis of the heat-treated CaPs powders demonstrated that increase in Zn^2+^ and Mg^2+^ substitution levels leads to the stabilization of the *β*-TCP phase. The obtained results are in agreement with the findings of Bigi et al. [87,88], who reported that Zn^2+^ and Mg^2+^ ions promote the thermal conversion of HAp into *β*-TCP, since these ions prefer structures with available octahedral coordination sites characteristic for *β*-TCP. As reported by Ren et al. [89,90], smaller substituted ions such as Zn^2+^ and Mg^2+^ in the HAp structure lead to significant lattice strain, resulting in more defects that promote the HAp decomposition. *In vivo* studies have shown that HAp degrades too slowly, with residues still present after 24 months and interfering with new bone formation. In contrast, *β*-TCP degrades too rapidly to maintain adequate structural support for bone ingrowth. Therefore, combining these two mineralogical phases may yield a biomaterial with an optimal resorption rate, enabling new bone formation while still providing adequate structural support [[87], [88], [89], [90], [91], [92], [93], [94]]. Therefore, in the present study, the scaffold HAp_1MIX, composed of HAp and *β*-TCP, would in principle be ideal with respect to achieving an appropriate resorption rate. However, the undesired changes in surface properties observed in this biomaterial led to reduced cell attachment.

*β*-TCP is generally considered a highly bioactive and soluble phase and is often used for the development of biomaterials for bone tissue engineering applications [95]. However, the literature reports inconsistent findings regarding cell attachment and proliferation on *β*-TCP. While some studies show enhanced cell attachment with modifications of *β*-TCP as a biomaterial, *β*-TCP itself may not always support optimal cell behavior [[96], [97], [98]]. Tarafder et al. [99] prepared three different compositions of *β*-TCP and HAp, where the study focused on understanding the effect of composition variation as well as electrical polarization of these composites on early-stage osteoblast-cell adhesion, proliferation, and ECM formation capability. Irrespective of the surface charge, early-stage osteoblast interactions on the composites were observed with the increasing HAp percentage. Davison et al. [58] studied how changing the scale of surface architecture of *β*-TCP can influence cellular resorption. Surface topographical features of *β*-TCP were influenced on the submicron or micron-scale. On submicrostructured samples, osteoclasts survived, fused, differentiated, and resorbed the *β*-TCP substrate; however, on microstructured samples, osteoclast survival, fusion and biomarker expression of osteoclast differentiation decreased, without evidence of resorption. The attachment of osteoblasts and osteoclasts to surfaces depends on various factors including surface roughness/topography, surface wettability (hydrophilicity and hydrophobicity), biomaterial stiffness, chemistry, protein adsorption capacity, ζ-potential, etc. [100].

Therefore, in the present study, it was essential to investigate how variations in the phase composition of HAp and *β*-TCP influence the surface potential and wettability of the fabricated scaffolds. Present study indicates that surface properties are appropriate when the scaffold is composed of a pure HAp phase, while an increasing amount of *β*-TCP negatively affects cell attachment. Cell attachment with varying ζ-potential is influenced by the electrochemical properties of the cell surfaces and of the materials they interact with. ζ-potential is a measure of the electrostatic potential at the interface of the double layer of charges (respectively stationary and diffuse layers) surrounding a particle or cell in suspension, which affects how cells interact with them. However, cell attachment is influenced not only by ζ-potential, but also by the interfacial chemistry, which affects protein adsorption: the first process that occurs immediately after a biomaterial is implanted in the body [[101], [102], [103]]. The HAp phase exhibits slightly negative ζ-potential values of −9.58 mV when sintered at 800 and −6.47 mV when sintered at 1200 °C [104]. The reported values are in accordance with those observed in present study, where non-substituted HAp2 scaffold showed ζ-potential values of −11.28 ± 1.96 mV. Only one study was found where the ζ-potential of *β*-TCP was measured depending on sintering temperature, along the analysis of cell attachment behaviour [105]. The *β*-TCP sintered at 900 °C exhibited a significantly lower number of cells attached to surface than *β*-TCP sintered at 1000, 1100, 1150, and 1200 °C. The ζ-potential of *β*-TCP sintered at 900 °C showed a highly negative charge (−13.8 mV) compared with the other sintering temperatures of 1000 (−4.0 mV), 1100 (−2.3 mV), 1150 (−3.5 mV), and 1200 °C (−3.6 mV). In the present study, the obtained biomaterials with increasing content of *β*-TCP demonstrated more negative ζ-potential values with reduced cell attachment. Obtained results are not in accordance with the study by Tanimoto et al. [105], indicating that substituted ions might play a significant role in surface properties. The surface chemistry of *β*-TCP is not fully understood and likely depends on various factors and preparation methods, which can lead to unpredictable clinical outcomes. Therefore, more efforts on understanding of *β*-TCP surface properties and interactions with cells need to be employed. In addition to its negative ζ-potential, which can influence cell attachment, HAp exhibits hydrophilic surface properties, whereas *β*-TCP tends to be more hydrophobic. This difference in wettability further impact cellular behaviour on the scaffold surface. Study by Brennan et al. [106] is in agreement with results observed in present study, where porous scaffolds based on HAp were highly hydrophilic and displayed immediate water adsorption, not allowing contact angle measurement, while *β*-TCP scaffolds took ∼2 h to completely absorb a water droplet. Therefore, in the scaffolds developed in this study, when ion substitutions were employed, the required sintering temperatures significantly impacted the phase composition and surface properties of the scaffolds. Ion substitutions and high sintering temperatures enhanced the crystallinity and phase purity of *β*-TCP, which further influence the hydrophobicity of the biomaterial. Electrostatic forces at the cell interface affect the nature of cell adhesion and function; but there is still limited knowledge about the impact of positive or negative surface charges on cell-material interactions [107]. Different effects of ζ-potential of biomaterials on cell adhesion are reported in literature, indicating complexity of the effect of surface properties on cell attachment [107,108]. To assess cell behaviour on materials with different ζ-potential, Gruening et al. [107] obtained titanium surfaces with ζ-potentials ranging from −90 mV to +50 mV by functionalizing them with various chemical groups. A significant enhancement of intracellular calcium mobilization was achieved on surfaces with a moderately positive ζ-potential (+1 to +10 mV) compared surfaces with a negative ζ-potential (−90 to −3 mV). Significant losses of cell activity were reported on surfaces with a highly positive ζ-potential (+50 mV) [107]. The study by Gruening et al. [102] provides valuable insights into the effect of ζ-potential on cellular behavior.

Along with ζ-potential and wettability (hydrophilicity/hydrophobicity), surface roughness also plays an important role in cell attachment. Therefore, multiple material properties collectively regulate the cell-attachment process. In our previous study [20], no noticeable differences in surface roughness were observed among the HAp, HAp_1MIX, and HAp_5MIX samples sintered at different temperatures based on SEM imaging; however, more suitable analytical techniques for quantifying surface roughness were not employed. However, in the present study it was demonstrated that lower sintering temperatures resulted in improved cell attachment. This is consistent with the well-established observation that increasing the sintering temperature reduces surface roughness, which in turn can negatively affect initial cell adhesion. In study by Ahn et al. it was found that adipose-derived stem cells adhered better to highly hydrophilic and rough surfaces and showed broadly stretched morphology compared with that on hydrophobic and smooth surfaces [109,110]. As there are limited numbers of the studies focused on examining differences of surface properties between different CaP phases, comprehensive studies need to be obtained to deepen our understanding of how surface charge, material composition, wettability and surface roughness influence cell–material interactions. It is important to understand the effect of each material characteristic individually, as well as the synergistic effect from their combined interaction as their effects are interconnected.

**Fig. 9:**
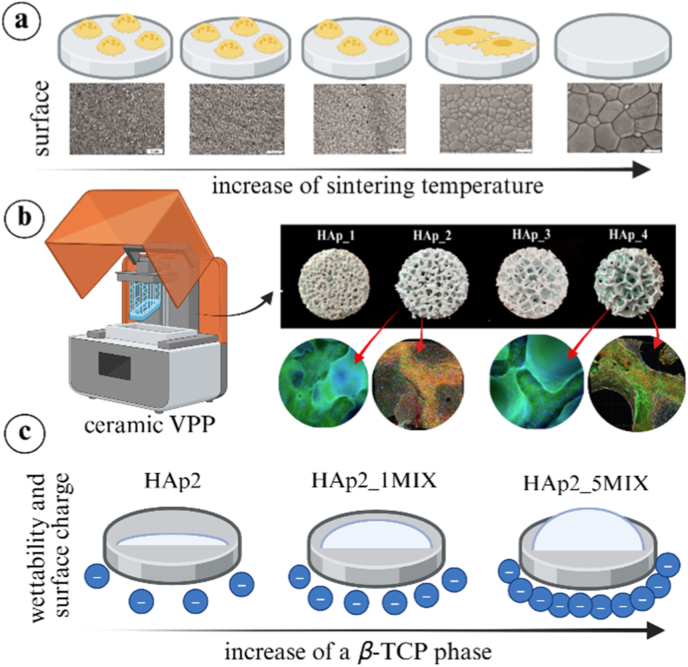
Schematic illustration of key finding of present and our previous work: (a) enhanced osteoclast adhesion on HAp sintered at lower temperatures within the investigated range (surface microstructure adapted from Ref. [20]); (b) the HAp2 scaffold exhibited the highest compressive strength and an appropriate cellular response, with cells and gene expression observed within the scaffold voids; and (c) wettability and surface charge were influenced by the mineralogical phases present, thereby impacting cell attachment. Created with Biorender.com.

Key findings of a present work are summarized in Fig. 9. The present study has several limitations that should be acknowledged: First, the biological experiments were conducted using cells from two donors and therefore inter-donor variability may influence the observed cellular responses. To ensure reliability of obtained results, we validated the mesenchymal origin and stemness of the cells prior to use by confirming the characteristic surface marker profile of undifferentiated hBMSCs and verifying multipotency through osteogenic (Supplementary Tables 1 and 2) and differentiation assays (Supplementary Figs. 1 and 2). For each condition, two independent wells were analyzed, and all analyses were performed with multiple replicates to confirm the reproducibility and reliability of the findings. Another limitation is that the mechanical properties of the scaffolds remain lower than those of cortical bone, restricting their use in load-bearing applications. Nevertheless, the mechanical properties fall within the range typically reported for porous trabecular bone. In addition, the material contains multiple CaP phases, which may lead to differences in degradation and resorption behaviour, as each phase exhibits distinct dissolution kinetics.

## 5 Conclusion

Inspired by the characteristics of natural bone microstructure and chemical composition, biomimetic scaffolds were developed using ceramic VPP fabrication technology. The obtained findings collectively support the osteoconductive potential of the tested scaffolds based on HAp and their suitability for bone tissue engineering applications. Scaffolds based on HAp both support new bone formation by osteoblasts and can be resorbed by osteoclasts. Scaffold HAp2, with an average pore size of ∼400 μm and porosity of 45.61%, has appropriate mechanical properties, microstructural design containing both small and large pore sizes to ensure efficient nutrient transport, allow penetration of cells into the scaffold to form new bone tissue, and support cell-cell interactions and osteogenic differentiation. When multi-substituted HAp was used for scaffold fabrication by the ceramic VPP method, the required debinding and sintering temperatures led to the transformation of HAp phase to *β*-TCP. This resulted in different surface properties with more negative surface potential, which in turn led to poor cell attachment on these scaffold types. In our previous study, we confirmed the positive effect of the substituted ions on the osteogenic properties. However, to better understand cell-biomaterial interactions, our future studies will focus on understanding the relationship between protein adsorption and cell attachment on biomaterials based on different CaPs, treated at different temperatures, and substituted with different ions. This aims to better understand these relationships and further open possibilities for scaffold development using ceramic VPP fabrication technology, ultimately enabling the development of personalized scaffold technologies for bone regeneration. Future work will be focus on improving the mechanical properties of the scaffolds and on systematically evaluating how the presence and distribution of different CaP phases influence the overall degradation and resorption rates.

## Funding

This project has received funding from the European Union's Horizon Europe research and innovation program under the 10.13039/100010665Marie Skłodowska-Curie grant agreement No. 101062225. The authors acknowledge the financial support from the 10.13039/501100002341Research Council of Finland (336666 S.M., 326588 S.M., 312413 S.M., 337607 S.M.).

## CRediT authorship contribution statement

**Antonia Ressler:** Conceptualization, Data curation, Formal analysis, Funding acquisition, Investigation, Resources, Validation, Writing – original draft, Writing – review & editing. **Roope Ohlsbom:** Conceptualization, Formal analysis, Investigation, Validation, Writing – original draft. **Virginia Alessandra Gobbo:** Formal analysis, Investigation, Writing – review & editing. **Markus Hannula:** Formal analysis, Investigation, Writing – review & editing. **Katharina Keck:** Formal analysis, Investigation. **Harish Swaminathan:** Formal analysis, Investigation, Validation. **Toni-Karri Pakarinen:** Resources. **Mehdi Mohammadi:** Formal analysis, Investigation. **Jari Hyttinen:** Investigation, Writing – review & editing. **Jonathan Massera:** Resources, Writing – review & editing. **Martin Schwentenwein:** Resources, Writing – review & editing. **Erkka J. Frankberg:** Funding acquisition, Resources, Writing – review & editing. **Erkki Levänen:** Funding acquisition, Resources, Supervision, Writing – review & editing. **Arjen Gebraad:** Conceptualization, Formal analysis, Investigation, Methodology, Supervision, Validation, Writing – review & editing. **Susanna Miettinen:** Funding acquisition, Resources, Supervision, Writing – review & editing.

## Declaration of competing interest

The authors declare that they have no known competing financial interests or personal relationships that could have appeared to influence the work reported in this paper.

## Data Availability

Data is available at online database listed in the manuscript.
