# Supplementary material for: Biomimetic bone calcium phosphate-based scaffolds fabricated via ceramic vat photopolymerization: Effect of porosity, sintering temperature, mineralogical phases and trace elements on the osteogenic potential
Source: Mater Today Bio. 2026 Mar 26;38:103074. doi: 10.1016/j.mtbio.2026.103074 (PMC13068806; doi:10.1016/j.mtbio.2026.103074)
Supplement: Multimedia component 1 [file mmc1.docx]

**Supporting information**

**Supplementary Table 1.** Surface marker expression undifferentiated hBMSCs derived from donor 1.

| **Antigen** | **Surface protein** | **Expression** | **Fluorophore** | **Manufacturer** |
| --- | --- | --- | --- | --- |
|  |  |  |  |  |
| CD14 | Serum lipopolysaccharide  binding protein | 1.9 | phycoerythrin-cyanine (PECy7) | BD Biosciences, Franklin Lakes, NJ, USA |
| CD19 | B lymphocyte-lineage  differentiation antigen | 1.5 | PECy7 | BD Biosciences |
| CD34 | Sialomucin-like adhesion molecule | 2 | allophycocyanin (APC) | Immunotools, Friesoythe, Germany |
| CD45 | Leukocyte common antigen | 1.2 | APC | BD Biosciences |
| CD54 | Intercellular adhesion molecule 1 | 16.5 | fluorescein isothiocyanate | BD Biosciences |
| CD73 | Ecto-5’-nucleotidase | 99.9 | phycoerythrin (PE) | BD Biosciences |
| CD90 | Thy-1 (T cell surface glycoprotein) | 99.9 | APC | BD Biosciences |
| CD105 | SH-2, endoglin | 99.8 | PE | R&D Systems, Minneapolis, MN, USA |
| HLA-DR | Major histocompatibility  class II antigens | 2.2 | PE | Immunotools |

**Supplementary Table 2.** Surface marker expression undifferentiated hBMSCs derived from donor 2.

| **Antigen** | **Surface protein** | **Expression** | **Fluorophore** | **Manufacturer** |
| --- | --- | --- | --- | --- |
| CD14 | Serum lipopolysaccharide  binding protein | 24.7 | phycoerythrin-cyanine (PECy7) | BD Biosciences, Franklin Lakes, NJ, USA |
| CD19 | B lymphocyte-lineage  differentiation antigen | 10.2 | PECy7 | BD Biosciences |
| CD34 | Sialomucin-like adhesion molecule | 2.6 | allophycocyanin (APC) | Immunotools, Friesoythe, Germany |
| CD45 | Leukocyte common antigen | 4.6 | APC | BD Biosciences |
| CD54 | Intercellular adhesion molecule 1 | 31.0 | fluorescein isothiocyanate | BD Biosciences |
| CD73 | Ecto-5’-nucleotidase | 100 | phycoerythrin (PE) | BD Biosciences |
| CD90 | Thy-1 (T cell surface glycoprotein) | 99.9 | APC | BD Biosciences |
| CD105 | SH-2, endoglin | 99.9 | PE | R&D Systems, Minneapolis, MN, USA |
| HLA-DR | Major histocompatibility  class II antigens | 5.0 | PE | Immunotools |


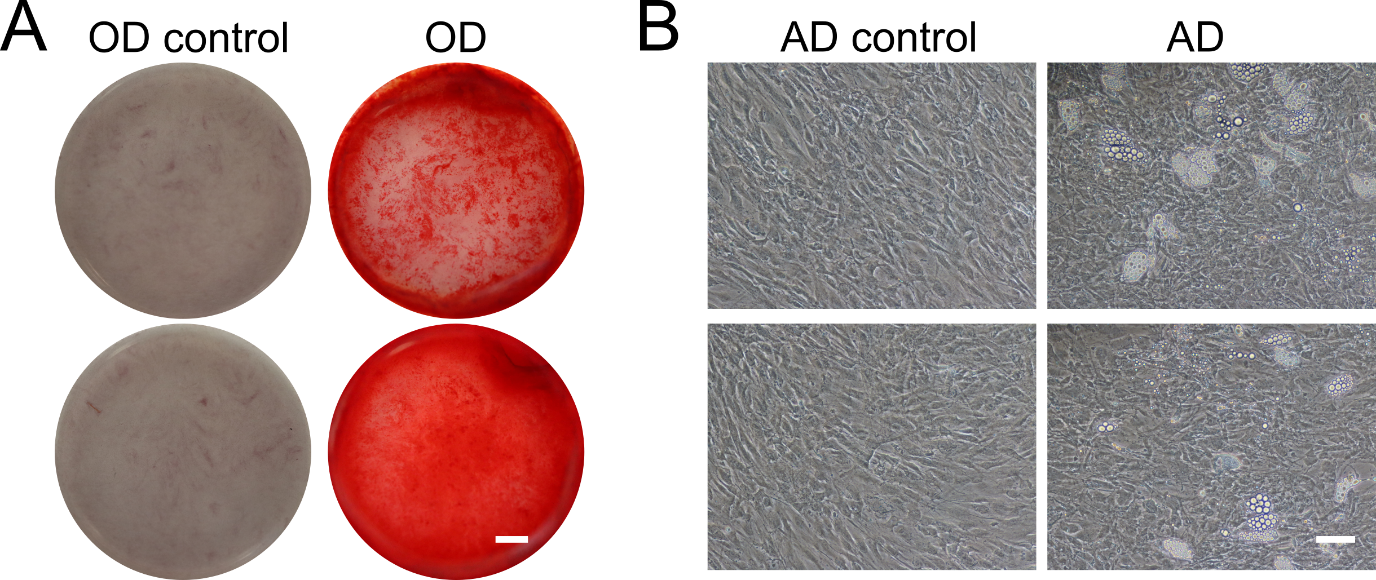


**Supplementary Figure 1.** Osteogenic and adipogenic differentiation potential of hBMSCs derived from donor 1. **(A)** Osteogenic differentiation (OD) of hBMSCs was assessed with Alizarin Red S staining of calcium phosphate mineral, after 21 days of culture. Scale bar represents ~ 200 mm. **(B)** Adipogenic differentiation (AD) was assessed by detecting the presence of lipid droplets with phase contrast microscopy, after 21 days of culture. Scale bar represents 100 µm. For all the culture conditions results are shown for two different culture wells.


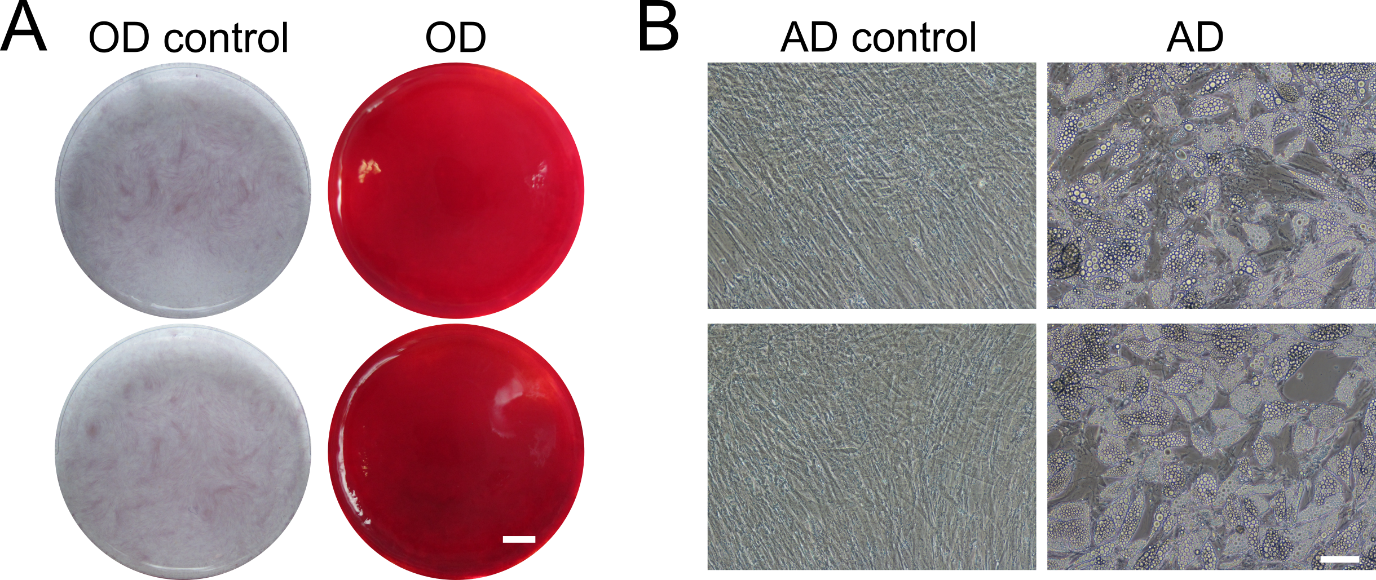


**Supplementary Figure 2.** Osteogenic and adipogenic differentiation potential of hBMSCs derived from donor 2. **(A)** Osteogenic differentiation (OD) of hBMSCs was assessed with Alizarin Red S staining of calcium phosphate mineral, after 21 days of culture. Scale bar represents ~ 200 mm. **(B)** Adipogenic differentiation (AD) was assessed by detecting the presence of lipid droplets with phase contrast microscopy, after 14 days of culture. Scale bar represents 100 µm. For all the culture conditions results are shown for two different culture wells.
